# How Does Allium Leafy Parts Metabolome Differ in Context to Edible or Inedible Taxa? Case Study in Seven Allium Species as Analyzed Using MS-Based Metabolomics

**DOI:** 10.3390/metabo13010018

**Published:** 2022-12-22

**Authors:** Mostafa H. Baky, Samir N. Shamma, Mohamed R. Khalifa, Mohamed A. Farag

**Affiliations:** 1 Department of Pharmacognosy, Faculty of Pharmacy, Egyptian Russian University, Badr City 11829, Egypt; 2 Institute of Global Health and Human Ecology, School of Sciences and Engineering, The American University in Cairo, P.O. Box 74, New Cairo 11835, Egypt; 3 Pharmacognosy Department, College of Pharmacy, Cairo University, Cairo 11562, Egypt

**Keywords:** Allium, volatiles, HS-SPME, GC–MS, chemometrics, metabolomics

## Abstract

Genus *Allium* (F. Amaryllidaceae) includes a wide variety of edible foods widely consumed for their nutritive as well as health benefits. Seven Allium species, viz., chives, Egyptian leek, French leek, red garlic, white garlic, red onion, and white onion aerial parts were assessed for metabolome heterogeneity targeting both aroma and nutrients phytochemicals. A headspace solid-phase microextraction (HS-SPME) and gas chromatography–mass spectrometry (GC–MS) were employed. Results revealed extensive variation in volatiles and nutrients profile among the seven Allium species represented by a total of 77 nutrients and 148 volatiles. Among edible Allium species, French leek encompassed high levels of nutrients, viz., sugars, fatty acids/esters, organic acids, and amino acids, compared to Egyptian leek. Sulfur aroma compounds appeared as the most discriminatory among Allium, taxa accounting for its distinct flavor. Furthermore, chemometric analysis of both datasets showed clear discrimination of the seven Allium species according to several key novel markers. This study provides the first comparative approach between edible and inedible aerial leafy parts of Allium species providing novel insight into their use as functional foods based on such holistic profiling.

## 1. Introduction

Recently, the higher growth in consumer demand for plant-based functional foods rich in bioactive metabolites with substantial health benefits warrants more progress in analytical tools that help in assuring their quality [1] and further as a tool for the discovery of less-explored nutraceuticals. Culinary herbs are widely consumed in the human diet either fresh or dry owing to their special aroma as well as several health benefits [2]. Allium is a well-known genus belonging to the family Amaryllidaceae, comprising about 900 species of herbs and vegetables [3]. Allium species are widely used in traditional medicine for treating several ailments such as headaches, colds, heart problems, and tumors, and for boosting immunity [4]. Allium health benefits are attributed to their richness in minerals, vitamins, essential amino acids, phenolics, and most importantly, organosulfur compounds (OSCs) [3]. Allium vegetables are characterized by their pungent aroma owing to their richness in OSCs specially S-alk(en)yl-L-cysteine sulphoxide derivatives which decompose upon thermal treatment as in cooking to yield simple thiosulfonates and sulfides [4,5].

OSCs are the most important bioactive metabolites in Allium species such as allyl cysteine derivatives, S-alk(en)yl-L-cysteine sulfoxides, thiosulfinates, thiosulfonates, and sulfides [4]. Several biological effects were reported for sulfur compounds, viz., antimicrobial, antihyperlipidemic, anti-inflammatory, immunomodulatory, antioxidant, hepatoprotective, and neuroprotective properties [6]. Owing to their activity in treating obesity and other metabolic disorders, viz., mild hypertension, hyperlipidemia, elevated blood glucose levels, and insulin resistance, Allium plants are effective as cardioprotective agents [7]. Several plants belonging to that genus are reported for their economic and medicinal values such as *A. cepa* L. (onion), *A. sativum* L. (garlic), *A. ampeloprasum* var. *kurrat* L. (Egyptian Leek), *A. ampeloprasum* var. *porrum* L. (French Leek), and *A. schoenoprasum* L. (chives) [8].

Garlic is considered an important source of beneficial minerals among Allium, including selenium, in addition to phytonutrients, i.e., flavonoids and OSCs [5]. Owing to its potential for reducing serum lipids and increasing beneficial HDL, garlic is commonly prescribed as antihyperlipidemic, antihypercholesterolemic, and antihypertensive [9]. Alliin (S-allyl-L-cysteine sulfoxide), the most abundant OSC in garlic, is hydrolyzed by an alliinase enzyme in crushed garlic to yield allicin (diallyl thiosulfinate), which is rapidly converted to diallyl disulfide [4]. Likewise, onion is rich in flavonoids, such as quercetin and OSCs mainly isoalliin (S-propenyl-L-cysteine sulfoxide), methiin (S-methyl-L-cysteine sulfoxide), and propiin (S-propyl-L-cysteine sulfoxide), converted to dipropyl thiosulfinate by the action of allinase [5]. Dipropyl thiosulfinate is more stable than allicin and can be further transformed into propyl disulfide, dipropyl disulfide, and propyl-propane thiosulfonate [3]. Egyptian leek (*A. ampeloprasum* var. *kurrat* L.), commonly known as kurrat, is one of the most widely consumed vegetables worldwide owing to its richness in nitrates, flavonoids, polysaccharides, and OSCs. The high OSC levels in leek can aid in reducing prostate, colorectal, stomach, and breast cancer risk as well as prevent atherosclerosis [10]. French leek (*A. ampeloprasum* var. *porrum* L.) is widely used worldwide in food recipes and possesses antibacterial, antifungal, and anticancer effects [11]. Moreover, chive (*A. schoenoprasum* L.), a less-common bulb-forming herb was reported for its milder flavor than other Alliums and is medicinally active, being antimicrobial and antihypertensive [12]. 

Owing to Allium consumption, their quality characteristics as green vegetables, especially their nutritional and sensory attributes, are important criteria to be measured. Recently, the application of the metabolome-based profiling of food targeting a large pool of chemicals is rapidly increasing to insure the quality of vegetables and fruits [13]. The abundance of volatile metabolites in food products is an important parameter for assessing their freshness and quality [1], especially considering consumer preferences toward such flavors in Allium vegetables. Gas chromatography coupled with mass spectrometry (GC–MS) is an important metabolomics tool used for the analysis of both volatiles and nutrient metabolites later, post-silylation [14]. Solid-phase microextraction (SPME) is an efficient tool used for the extraction of volatiles by enhancing their adsorption on a fused-silica fiber avoiding their decomposition [2]. Owing to the generation of huge complex data from MS-based analysis, multivariate data analyses can aid in the data visualization of such a complex dataset. Unsupervised principal component analysis (PCA) and supervised orthogonal projection to least squares discriminant analysis (OPLS-DA) are the most common tools that aid in sample segregation and biomarker recognition [1]. 

Several studies were reported on Allium species metabolites profiling mostly focused on sulphur compounds and in the context of their biological activities [15,16,17], there is a deficiency in comparative studies between edible and inedible Allium vegetables. To the best of our knowledge, this is the first comparative report to assess heterogeneity in nutrients and aroma compounds in edible versus inedible Allium species cultivated in Egypt. In this study, seven major Allium species of the same origin were compared using HS-SPME-GC–MS and GC–MS post-silylation, coupled with multivariate data analysis. Vegetables examined include aerial parts from edible Allium, viz., red onion, white onion, Egyptian leek, and French leek in addition to inedible ones, viz., red garlic, white garlic, and chives assessed for variation in their nutritive and aroma metabolites. Such an approach shall aid in comparing inedible Alliums and identify potential uses compared to more explored ones, and likewise, aid to identify better markers for their sensory and nutrient attributes to be targeted in future breeding or quality control studies of the Allium genus.

## 2. Materials and Methods

### 2.1. Plant Material

Seven Allium species aerial parts, viz., *A. cepa L.* (red onion), *A. cepa* var. *cepa* L. (white onion), *A. sativum* L. (red garlic), *A. sativum* var. *sativum* L. (white garlic), A. *ampeloprasum* var. *kurrat* L. (Egyptian leek), *A. ampeloprasum* var. *porrum* L. (French leek), and *A. schoenoprasum* L. (chives) were collected from local farms in Qalyub, El-Qalyubia Governorate, Egypt, during April and May 2021; all plant names that follow are listed in the plant list website (http://www.theplantlist.org/) accessed on 1 April 2021. The fresh aerial parts were lyophilized and kept in the freezer at −20 °C till preparation for GC–MS analysis. A voucher specimen from each plant was deposited at the College of Pharmacy Herbarium, Cairo University, Cairo, Egypt. 

### 2.2. SPME and Chemicals

Fibers used in SPME volatile extraction are stable, flex-coated with divinylbenzene/carboxen/polydimethylsiloxane (DVB/CAR/PDMS, 50/30 µm), or PDMS (polydimethylsiloxane), and were purchased from Supelco (Oakville, ON, Canada). Volatile standards, amino acids, fatty acids, and sugars were provided by Sigma Aldrich (St. Louis, MO, USA).

### 2.3. GC–MS Analysis of Silylated Primary Metabolites

Freeze-dried finely powdered aerial parts (100 mg) were extracted with 5 mL 100% methanol with sonication for 30 min and frequent vortex shaking. Three different samples from each Allium aerial parts accession were analyzed under the same conditions to evaluate biological replicates. Methanol extract (100 µL) was aliquoted in screw cap vials and left to evaporate under a nitrogen gas stream until complete dryness. For derivatization, 150 µL of N-methyl- N-(trimethylsilyl)-trifluoroacetamide (MSTFA) previously diluted 1/1% with anhydrous pyridine was mixed with the dried methanol extract and incubated for 45 min at 60 °C prior to analysis using GC–MS. Separation of silylated derivatives was achieved on an Rtx-5MS (30-m length, 0.25-mm inner diameter, and 0.25-m film). Quantitative analysis of the primary metabolites followed the detailed protocol previously published in [18]. Soluble sugars, amino acids, organic acids, and fatty acids were quantified using standard curves of glucose, glycine, citric and stearic acids, and results were expressed as mg/g. Four serial dilutions were prepared from 10 to 600 ug/mL for establishing the standard curves. Calibration curves for glucose, glycine, citric acid, and stearic acids displayed ca. 0.99 correlation coefficient. 

### 2.4. SPME–GC–MS Volatiles Analysis

Freeze-dried finely powdered leaves (100 mg) were placed in SPME screw cap vials (1.5 mL) spiked with 10 µg (Z)-3-hexenyl acetate with fibers inserted manually above, and placed in an oven kept at 50 °C for 30 min. HS-SPME analysis of the volatile compounds was performed as reported in [1] with slight modifications. The fiber was subsequently withdrawn into the needle and then injected manually into the injection port of a gas chromatography–mass spectrometer (GC–MS). GC–MS analysis was adopted on an Agilent 5977B GC/MSD equipped with a DB-5 column (30 m × 0.25 mm i.d. × 0.25 µm film thickness; Supelco) and coupled with a quadrupole mass spectrometer. The interface and the injector temperatures were both set at 220 °C. Volatile elution was carried out using the following gradient temperature program: oven was set at 40 °C for 3 min, then increased to 180 °C at a rate of 12 °C/min, kept at 180 °C for 5 min, and finally increased at a rate of 40 °C/min to 240 °C, and kept at this temperature for 5 min. Helium was utilized as a carrier gas with a total flow rate of 0.9 mL/min. For ensuring the complete elution of volatiles, SPME fiber was prepared for the next analysis by placing it in the injection port at 220 °C for 2 min. For assessment of biological replicates, three different samples for each accession were analyzed under the same conditions. Blank runs were made during sample analyses. The mass spectrometer was adjusted to EI mode at 70 eV with a scan range set at m/z 40–500.

### 2.5. Metabolites Identification and Multivariate Data Analyses

Identification of both volatile and silylated components was performed by comparing their retention indices (RI) in relation to n-alkanes (C6–C20), mass matching to NIST, WILEY library database, and with standards if available. Peaks were first deconvoluted using AMDIS software (accessed on 1 April 2021, www.amdis.net) [19] before mass spectral matching. Peak abundance data were exported for multivariate data analysis by extraction using MS-Dial software under same conditions cited in [20]. Data were then subjected to principal component analysis (PCA), hierarchical clustering analysis (HCA), and partial least squares discriminant analysis (OPLS-DA) using SIMCA-P version 13.0 software package (Umetrics, Umeå, Sweden). Markers were subsequently identified by analyzing the S-plot, which was declared with covariance (p) and correlation (pcor). All variables were mean-centered and scaled to Pareto variance. Model validation was assessed by computing the diagnostic indices, viz., Q2 and R2 values, p-value, and permutation testing.

## 3. Results and Discussion

The main goal of the current study was to assess metabolite variations in seven Allium species’ aerial parts collected from Egypt in the context of their nutrient and aroma profiles using an MS-based metabolomics approach. Three independent biological replicates were concurrently analyzed by GC–MS for best assessment in metabolite variations. Aerial parts from edible, i.e., red onion, white onion, French leek, and Egyptian leek versus inedible, i.e., red garlic, white garlic, and chives were included for comparison in metabolite composition and potential uses based on such holistic chemical profile.

### 3.1. Primary Metabolites Profiling in Allium Species Aerial Parts via GC–MS Analysis (Post-Silylation)

**Figure 1:**
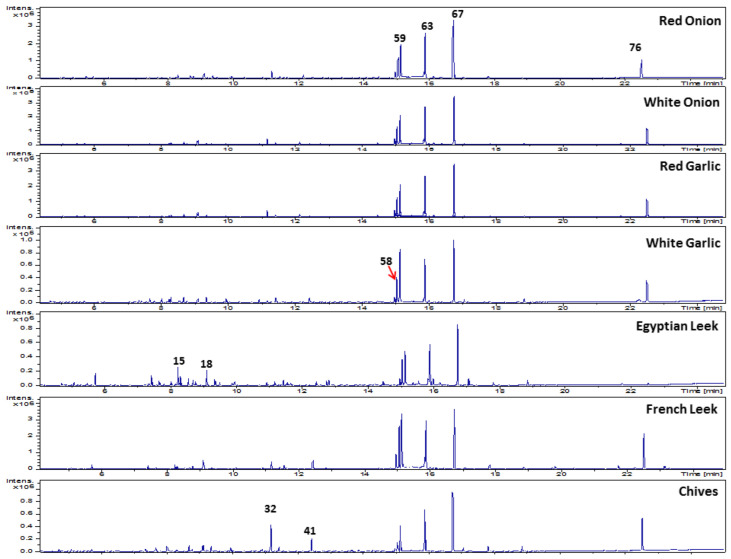
Representative GC–MS chromatograms of primary metabolites were identified in seven Allium species post-silylation. The corresponding compound names for numbered peaks follow those listed in Table 1: 15, L-Leucine; 18, Glyceric acid; 32, Malic acid; 41, D-Psicose; 58, D-Tagatofuranose; 59, D-Fructopyranose; 63, D-Manopyranose; 67, D-Glucopyranose; 76, Sucrose.

**Figure 2:**
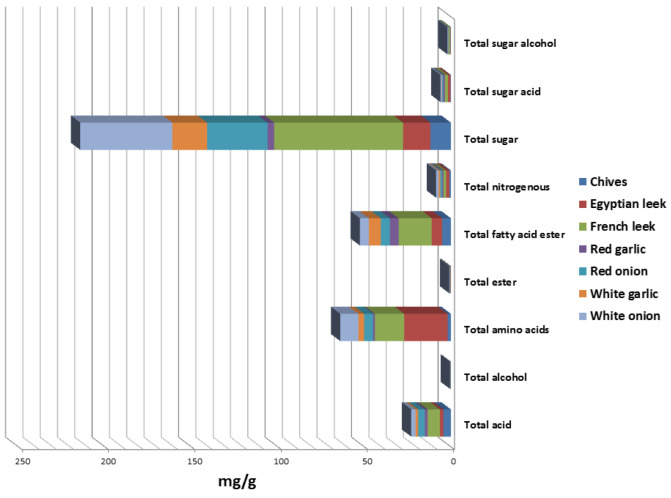
Different classes of identified primary metabolites quantified as mg/g in seven Allium species.

Post-silylation GC–MS analysis was employed to assess variation in primary nutritive metabolites among Allium species. A total of 77 peaks (Table 1, Figure 1) were annotated, including sugars (mono- and disaccharides), sugar alcohols, sugar acids, fatty acids/esters, organic acids, amino acids, nitrogenous compounds, and alcohols (Figure 2). 

#### 3.1.1. Sugars

Sugars were detected as the most dominant primary metabolites represented by 17 peaks in all Allium species. The highest levels of sugars represented by monosaccharides were detected in French leek, white onion, red onion, white garlic, and Egyptian leek, amounting to ca. 74.5, 53.2, 34.9, 20.04, and 15.7 mg/g, respectively. In contrast, lower sugar levels were detected in inedible chives and red garlic at ca. 11.8 and 3.8 mg/g, respectively. Such variations in sugar levels indicate that the inedible parts are less palatable compared to edible parts being low in sugars. Glucose (peaks 64, 67) was detected as the main sugar found at much higher levels in edible Allium, viz., white onion, French leek, red onion, and Egyptian leek at 17.3, 16.3, 11.2, and 5.2 mg/g, respectively. Among inedible Allium, only white garlic showed levels comparable to that of leek at 5.4 mg/g. Likewise, fructose (peak 59) was detected at higher levels in edible Allium species at ca. 16.2, 10.7, 6.4, and 3.8 mg/g in French leek, white onion, red onion, and Egyptian leek, respectively. Likewise, mannose (peak 63) was detected at higher levels in edible Allium, viz., white onion, French leek, and red onion at 13.6, 12.7, and 8.6 mg/g, respectively. Interestingly, and within the same vegetable, sucrose (peak 76) as a major disaccharide was detected at much a higher level in French leek at 10.3 mg/g versus trace levels in Egyptian leek. 

Compared to free sugars, sugar alcohols were detected at trace levels in all Allium species represented by erythritol (peak 34), threitol (peak 38), mannitol (peak 66), and myoinositol (peak 70). Likewise, sugar acids were detected at much lower levels in all Allium species ranging from 0.2–1.8 mg/g, represented by glyceric acid (peak 18) and L-threonic acid (peak 40). 

#### 3.1.2. Amino Acids/Nitrogenous Compounds

Amino acids were detected generally at comparable higher levels in edible Allium species 25.1, 16.9, 10.5, and 5.1 mg/g in Egyptian leek, French leek, white onion, and red onion, respectively. Such higher amino acid levels among edible Alliums rationalize their use as functional foods with substantial nutritional value. L-leucine (peak 15) was detected as the most abundant amino acid in Egyptian leek, French leek, and white onion at ca. 5.3, 3.2, and 1.4 mg/g, respectively. Valine (peak 10) and alanine (peak 7) were detected in edible Alliums such as Egyptian leek, French leek, and white onion at comparative levels ranging from 1–3.5 and 1.8–2.6 mg/g, respectively. Owing to their nutritional value, amino acids play a pivotal role in human health as an anti-oxidative response, metabolic regulation, and immune response [21]. Being rich in leucine and valine, edible Allium species are considered a good source of such nutritionally important essential amino acids, compared to major consumed food sources of leucine and valine such as meat (39.9%), grain products (22.1%), and dairy products (20.0%) as well as other amino acids such as glutamic acid, tyrosine, lysine, glycine, leucine, isoleucine, phenylalanine, and threonine [22]. Proglutamic acid (peak 35) was abundant only in French leek at 4.1 mg/g, playing a role in enhancing protein function asides from its anti-proliferative and antimicrobial actions [23]. Nitrogenous compounds were detected at trace levels in all Allium species ranging from 0.7–1.9 mg/g represented by nicotinic acid (peak 13), uracil (peak 19), ethanolamine (peak 23), and aminobutyric acid (peak 37). As expected, thermolabile non-protein sulfur-containing amino acids, viz., S-methyl-L-cysteine sulfoxide, and S-methyl-L-cysteine were not detected as they rapidly decompose at the high operating temperature of GC/MS to yield sulfur compounds mainly dimethyl disulfide, dimethyl trisulfide, dimethyl thiosulfinate, and dimethyl thiosulfonate [24].

#### 3.1.3. Fatty Acids/Esters/Sterols

Fatty acids/esters were detected at relatively high levels in French leek at ca. 19.15 mg/g compared to levels ranging from 5.0–6.8 mg/g in other Allium species. Both saturated fatty acids and monounsaturated fatty acids were detected in all Allium species. Pamitic acid (peak 68) and stearic acid (peak 74) were detected as the major saturated fatty acids in Allium species at levels ranging from 1.6–2.4 and 1.9–2.5 mg/g, respectively. Oleic acid (peak 72), linoleic acid (peak 71), and α-linolenic acid (peak 73), as examples of monounsaturated and poly-unsaturated fatty acids with positive effects on heart health and atherosclerosis risk [19] were detected in all Allium species, though at low levels (0.1–0.4 mg/g) unlikely to exert a positive effect. Regarding fatty acyl esters, monostearin (peak 77) and 1-monopalmitin (peak 75) were detected at higher levels in French leek at 6.7 and 7.3 mg/g, respectively, compared to much lower levels in other Allium species, and suggestive that French leek was the most enriched in saturated lipids among examined taxa. 

#### 3.1.4. Organic Acids/Alcohols

Organic acids were detected in all Allium species at comparable levels between edible and inedible Allium species at ranges of 2.5–7.1 and 1.4–4.3 mg/g, respectively. Organic acids can improve digestion, help in protein utilization, and act as food preservatives [1]. Malic acid (peak 32), which accounts for the sour taste of fruits [25], was detected in all Allium species, especially French leek, chives, and red onion at ca. 3.5, 3.1, and 2.2 mg/g, respectively. Moreover, pyruvic acid, the major acid responsible for Allium pungency as a source of sulfur compound biosynthesis [16], was detected in all Allium species. Compared to acids, alcohols were detected at trace levels in all Allium species represented mainly by 1,3 propandiol (peak 2), diethylene glycol (peak 12), and 1-nonanol (peak 25). 

### 3.2. HCA and PCA Analysis of Post-Silylated Primary Metabolites Allium Species

**Figure 3:**
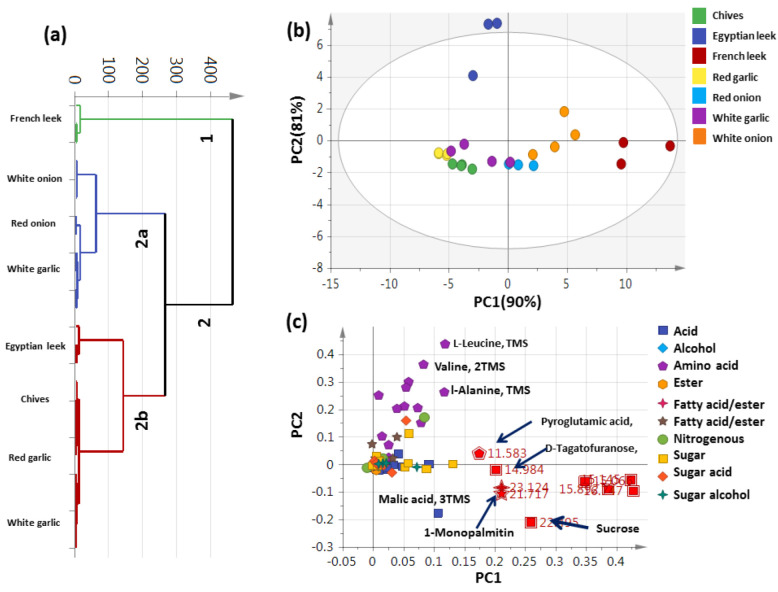
Unsupervised multivariate data analyses of the studied Allium species derived from the modeling of the silylated primary metabolites dataset via GC-MS post silylation (n = 3). (**a**) HCA plot. (**b**) PCA score plot of PC1 vs. PC2 scores. (**c**) The respective loading plot for PC1 and PC2, providing mass peaks and their assignments. The metabolome clusters are placed in two-dimensional space at the distinct locations defined by two vectors of principal component PC1 = 90% and PC2 = 81%.

Multivariate data analyses were employed including HCA and PCA analyses to assess variations in primary metabolites among the seven Allium species (Table 1). HCA depicted a dendrogram of two distinct clusters (Figure 3a), with French leek clustered separately in group 1, whereas other Allium species were divided into two subdivisions from group 2. The edible onion (white and red types) was clustered against the inedible white garlic in subgroup 2a. Moreover, Egyptian leek was clustered against chives and garlic (red and white) in subgroup 2b. However, the clustering of edible (red and white onion) and inedible Allium species (chives and red and white garlic) together in group 2 indicated that HCA cannot readily discriminate among species based on their silylated primary metabolites. A PCA model (Figure 3b) composed of two orthogonal PCs accounted for 90% of the total variance, with the segregation of all edible French leek specimens to the right side of PC1 with negative score values, whereas edible Egyptian leek was positioned towards the positive left side of PC1 and scattering of other samples at the middle axis. Such an unexpected clustering of leek specimens away from each other is due to the large variations in metabolite content, especially sugars, fatty acids/esters, and amino acids. Inspection of the corresponding loading plot in Figure 3c revealed that higher amino acid levels were detected in Egyptian leek, represented by L-leucine (peak 15) valine (peak 10), and L-alanine (peak 7), and to account for their segregation at the upper side. Moreover, the abundance of pyroglutamic acid (Peak 35), fructose (peak 59), tagatofuranose (peak 57), mannose (peak 63), and disaccharide sucrose (peak 76), malic acid (peak 32), and 1-monopalmitin (peak 75) were indicative of French leek, segregated towards the lower right side of the score plot. From the aforementioned models PCA plot and loading plots only two edible Allium samples were differentiated, viz., Egyptian leek and French leek from other Allium species.

### 3.3. OPLS-DA Analysis of Allium Aerial Parts 

Compared to the unsupervised multivariate model (PCA), supervised orthogonal partial least squares discriminant analysis (OPLS-DA) was adopted to better classify Allium samples, (Figure S1) by constructing three respective models of white onion, French leek, and Egyptian leek each modeled against the other six Allium species. OPLS-DA is a better way for identifying marker metabolites with great efficiency by selecting the most suitable variables for differentiation between two classes [19]. The French leek model showed higher prediction power with Q2 = 0.93 and R2 = 0.96 versus the Egyptian leek model at 0.86 and 0.90, and the white onion model at 0.73 and 0.88, respectively. The respective loading S-plot (Figure S1a) revealed that monosaccharides i.e., glucose, mannose, and fructose were abundant in white onion, with *p*-value < 0.000217456. Additionally, the loading S-plot (Figure S1e) revealed the abundance of lipids, viz., 1-monopalmitin, monostearin, and sugars, viz., sucrose, fructose, and tagatofuranose in French leek compared to other samples. Likewise, the loading S-plot (Figure S1f) revealed that amino acids, viz., valine and leucine were higher in Egyptian leek compared to other Allium species. The segregation of French from Egyptian leek as most distant in that model is unexpected and suggestive that primary metabolites cannot provide a strong model for Allium classification and warrant other metabolite markers for that taxa classification. These results are in agreement with our previous report on licorice root classification using GC/MS targeting its primary metabolites [26].

### 3.4. Headspace-SPME–GC–MS Volatiles Analysis of Allium Species

Allium volatiles, especially OSCs, are highly characteristic and responsible for that genus’s unique aroma and taste and consumer preferences for these vegetables posing them as potential markers to be exploited for Allium classification. A total of 148 volatile peaks were identified in 7 Allium aerial parts using headspace–solid-phase microextraction (HS-SPME) (Table S1, Figure S2). Allium-identified volatiles belonged to several classes., viz., sulfur compounds, organic acids/alcohols, aldehydes/ketones, aliphatic/aromatic hydrocarbons, esters, fatty acids/esters, furan, nitrogenous, phenols/ethers, and monoterpene hydrocarbon (Figures S3 and S4) as discussed in the next subsections.

#### 3.4.1. Sulfur Compounds

The genus Allium is enriched with OSCs, providing its characteristic onion/garlic-like odor after tissue disruption, and is responsible for Allium’s pungent spicy taste and further health benefits [27,28]. These volatile sulfur compounds are derived from sulfur non-protein amino acids, namely S-alk(en)ylcysteine S-oxides or N-oxides, which enzymatically get cleaved from disrupted tissues to yield a large number of sensory-active metabolites, including pungent thiosulfinates and lachrymatory sulfines [27]. Sulfur compounds were detected as the major volatiles in all Allium species, represented by 53 peaks and detected at higher levels in inedible Allium, viz., chives, white garlic, and red garlic at ca. 86.6, 78.06, and 63.1%, respectively, compared to edible white onion, red onion, Egyptian leek, and French leek at lower levels of 59.6, 46.5, 29.2, and 24.5%, respectively. These results suggest that chives and garlic leafy parts present a richer source of OSCs than other examined species. Tetrathiaoctane (peak 128) was detected as the major sulfur compound in chives at 63.5%, compared to very low levels in other Allium samples ranging from 0.4–1.9% and suggesting that it can serve as a marker for chives’ aerial parts. Tetrathiaoctane was previously detected in A. hooshidaryae, Scorodophloeus zenker (tropical garlic tree), and Afrostyrax lepidophyllus (bush onion seeds), and is responsible for garlic-like irritant odor [29,30]. Likewise, 2,4,5-trithiahexane was detected at high levels in chives only at 13.5% compared to traces in other Allium species. In addition, 2,4,5-Trithiahexane was previously detected in Marasmius alliaceus (Garlic Marasmius) [31] and Scorodophloeus borneensis (wood garlic) with antiplatelet aggregation activity [32]. Whether chive would exhibit similar antiplatelet aggregation should be examined considering its richness in OSCs. Another key OSC in Allium is diallyl disulfide (peak 60), detected at higher levels in inedible red and white garlic aerial parts at comparable levels of ca. 36.7 and 32.7%, respectively, and in accordance with previous reports [33,34], suggesting that both have similar aroma profiles. Diallyl disulfide, a major OSC compound in garlic formed by the degradation of allicin, is reported for its myriad biological activities as a potent antioxidant, hypolipidimic, anticancer, and as protection against CCl4-induced hepatotoxicity and cisplastin-induced nephrotoxicity [33,35]. In contrast, propyl disulfide (peak 69) was detected at much higher amounts in edible Allium, mostly in white and red onion, at similar levels of 18%, and detected at trace levels in chives and garlic. Propyl disulfide is a major OSC in A. cepa with potent antioxidant, anti-inflammatory, antihyperlipidimic, antimicrobial, and anticancer activity [36]. Moreover, propyl disulfide was detected in French and Egyptian leeks at much lower levels compared to onion specimens at 5.7 and 3.6%, respectively. Other OSCs that showed variation among different Allium species include allyl trisulfide, detected at higher levels in white and red garlic at ca. 15.5 and 4.5%, respectively. Compared to other previous studies, sulfides, disulfides, and trisulfides were detected as the most abundant OSCs (89–92%) in the volatile blend of onion flakes and onion rings with an abundance of dipropyl disulfide [37]. Moreover, the garlic bulb was reported to be enriched in OSCs, especially diallyl disulphide, diallyl sulphide, and diallyl trisulphide [38].

#### 3.4.2. Aldehydes/Ketones

Aldehydes/ketones were detected in the edible Allium aerial parts, amounting to 27.5, 22.8, 17.6, and 10.8% of the total VOCs in Egyptian leek, French leek, red onion, and white onion, respectively, compared to 18.7, 10.7, and 3.2% in red garlic, white garlic, and chives, respectively, as inedible Allium species. Benzaldehyde (peak 28) was detected as the major aldehyde in Egyptian and French leeks at 10.4 and 3.8%, respectively, compared to relatively trace levels in other Allium samples. Interestingly, although leek specimens showed diverse primary metabolite profiles, aroma analysis showed that these were close in composition. Benzaldehyde of bitter almond odor, with the medicinal values of being anti-inflammatory and antimicrobial, was previously detected in the volatile blend of several Allium species [37]. Several short-chain C6 aldehydes were detected, likely derived from fatty acids via the LOX pathway. Additionally, 2-Octenal (peak 54) was detected at a higher level in red garlic at 7.7% versus trace levels in all other Allium vegetables. A short-chain fatty aldehyde with green and fatty-like flavor [39], 2-Octenal was previously detected in A. tenuissimum [40]. A safe food additive with antimicrobial potential [1], 2-Hexenal (peak 6) was detected in all Allium species, especially in edible Allium species. Whether the abundance of antimicrobial VOCs such as 2-hexenal could account for the improved shelf life of certain Allium vegetables should be examined.

Ketones were represented in Allium species with several peaks such as 3,5-octadien-2-one (peak 58 and 62), an aroma compound detected in green vegetables and a marker of their freshness detected at higher levels in Egyptian leek at 4.8% compared to trace levels in other species. β-ionone (peak 126), an isoprenoid volatile compound derived from carotenoids with potential health benefits, viz., being anti-inflammatory and antimicrobial [1], was detected in Allium species, especially Egyptian and French leek at 1.3 and 1.7%, respectively. Profiling of carotenoids level using other techniques such as LC/MS should now follow considering their potential antioxidant actions.

#### 3.4.3. Alcohols

Alcohols represented by 11 peaks were detected at relatively high levels in edible Allium species, viz., French and Egyptian leek and red and white onion at ca. 11–13% and 5–9%, respectively, compared to 1.1–3.3% in all inedible Allium species. Phytol (139), a diterpene long-chain unsaturated acyclic alcohol, was the most abundant alcohol in edible Allium species, detected at high levels in red onion and French leek at ca. 4.5%. Phytol is characterized by its pleasant aroma and myriad biological activities [41], aside from serving as a precursor of vitamin E and contributing to its antioxidant effect. Further analysis of vitamin E levels would provide further evidence of such effects among Alliums based on that aroma profile using other techniques. Another green leaf volatile, 3-Hexenol (peak 7), emitted to protect against insect attached [42], was detected in all Allium species, especially in French and Egyptian leek at ca. 2.5%. 

#### 3.4.4. Acids/Esters and Furans

Compared to alcohols, acids were detected at relatively lower levels in edible Allium species aroma at ca. 8.4, 5.9, 4.5, and 2.9% in Egyptian leek, French leek, white onion, and red onion, respectively. However, being inedible, red garlic aerial parts contained considerable levels of acids at 5.01%. Short-chain acids were detected in Allium leafy parts, represented by pentatonic acid (peak 29) at higher levels in leek (3–5%), and heptanoic acid (peak 57) was detected in red garlic and white onion at ca. 3.6 and 2.3%, respectively. Other less abundant acids included isovaleric, tigilic, valeric, benzoic, caprylic, nonanoic, and n-capric acids. Unlike acids, esters were detected at trace levels in all Allium species at a range of 0.1–1.8% and represented by 4-hexenyl acetate (peak 44) and benzyl benzoate (peak 137). Volatile short-chain fatty acids and esters contribute to the overall aroma of fruits and vegetables [43], but unlikely in Allium aerial parts, which are more dominated by sulphur compounds, likewise in the bulb. Furans were detected at trace levels (0.06–0.63%) in all Allium aerial parts, represented by hydroxymethyfurfural (peak 92) and 2-octanoylfuran (peak 132), as expected, as these samples were not subjected to any thermal treatment.

#### 3.4.5. Aliphatic and Aromatic Hydrocarbons

Aromatic hydrocarbons were detected at relatively high levels in all edible Allium aerial parts at 10.0, 7.8, 3.9, and 3.5% in French leek, Egyptian leek, white onion, and red onion, respectively, compared to trace levels in inedible Alliums. Naphthalene (peak 84) was detected at relatively high levels in French and Egyptian leek at ca. 4%. In contrast, aliphatic hydrocarbons were detected at much lower levels in Allium aerial parts found at highest levels in white and red garlic at comparable levels of ca. 3%. 

#### 3.4.6. Fatty Acids/Esters

Fatty acids were detected at high levels in edible Allium aerial parts, compared to inedible species ranging from 7–15% in French leek, red onion, white onion, and Egyptian leek, respectively. Palmitic acid (peak 148) was the major saturated fatty acid detected in edible Allium aerial parts at 4–9%, with the highest level in French leek and red onion at 9.2 and 8.2%, respectively. These results were in accordance with silylated Allium samples which revealed the presence of palmitic acid (1.7–2.4%) and stearic acid (1.9–2.5%). The abundance of fatty acids in Allium was in accordance with previous studies, which revealed the detection of palmitic acid (20–23%) in garlic and onion bulbs [44]. Compared to palmitic acid, which is not regarded as having health benefits, palmetoleic acid (peak 47), a monounsaturated fatty acid with a positive effect on human health, such as being anti-inflammatory, hypolipidemic [45], was detected in edible Allium aerial parts though at much lower levels 0.4–1.4%. Fatty acid acyl esters were detected at trace levels in all Allium aerial parts represented mainly by methylpalmitate (peak 145), methyl laurate (peak 127), and methy tridecanoate (peak 135).

#### 3.4.7. Monoterpene Hydrocarbons

Monoterpenes were detected at comparable low levels amounting to 1.4–4.7% of the total volatile blend of Allium aerial parts, with the highest level in Egyptian leek at 4.7%. Monoterpene hydrocarbons were represented by nine peaks, especially α-phyllandrene (peak 32s and 56), o-cymene (peak 47), limonene (peak 49), and γ-terpinene (peak 55). Unlike monoterpene hydrocarbons, oxygenated forms were detected at trace levels (0.6–3.05%) in all Allium samples represented by estragole, arosol, and anethole. Anethole (peak 101) was detected in all Allium species, especially white and red onion, at ca. 2.8 and 1.2%, respectively. Anethole is a key aroma compound used as a flavoring agent in food, cosmetics, perfumery, and by pharmaceutical manufacturers in addition to myriad biological activities, viz., being antimicrobial and anti-inflammatory [1].

#### 3.4.8. Phenols/Ethers and Nitrogenous Compounds

Phenols/ethers represented by safrole (peak 102) and carvacrol (peak 104) were detected in all Allium samples, especially edible ones, at trace levels. Likewise, nitrogenous compounds were detected in all Allium aerial parts in trace levels (0.1–1.0%) represented by indole (peak 103). 

### 3.5. PCA Analysis of Allium Aroma Profile via HS-GC–MS

**Figure 4:**
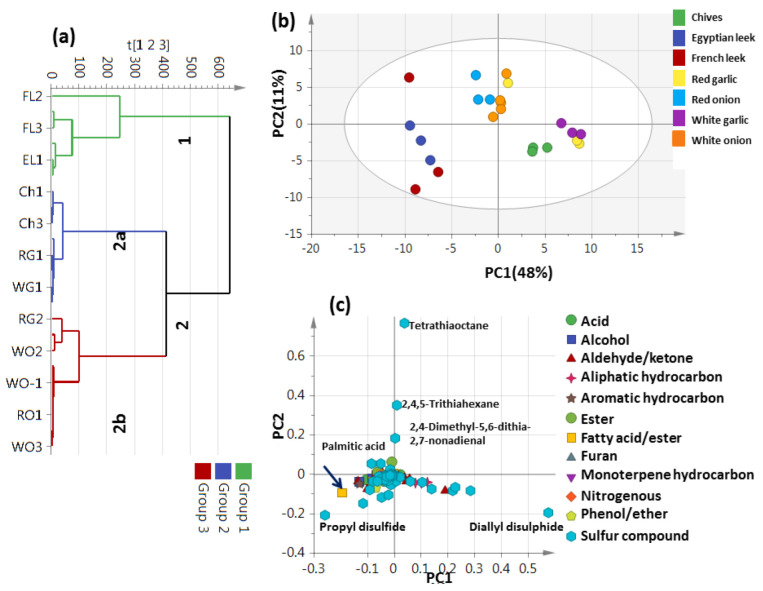
Unsupervised multivariate data analyses of the studied Allium species derived from modeling volatile metabolites dataset analyzed using SPME GC-MS (n = 3). (**a**) HCA plot. (**b**) PCA score plot of PC1 vs. PC2. (**c**) Loading plot for PC1 and PC2, providing variant peaks and their assignments. The metabolome clusters are placed in two-dimensional space at the distinct locations defined by PC1 = 48% and PC2 = 11%.

Distribution of aroma metabolites in Allium species aerial parts was assessed in a more holistic manner using HCA and PCA analyses to reveal how aroma datasets would differ compared to nutrient primary metabolite datasets (Figure 4). HCA depicted a dendrogram of three distinct clusters (Figure 4a): French and Egyptian leek were clustered in group 1, chives and red and white garlic were clustered in 2a, and red garlic and red and white onion were positioned into two subdivisions from group 2b. The clustering of edible and inedible Alliums together in group 2 indicated that HCA failed to characterize volatile heterogeneity among Allium species based on such stratification. A PCA model (Figure 4b) consisting of two orthogonal PCs accounted for 59% of the total variance, with the obvious discrimination of chives and red and white garlic clusters at the right side of PC1. In contrast, towards the left side of PC1 were two clusters, one for Egyptian and French leek and the other for red and white onion. The corresponding loading plot of Figure 4c revealed higher OSC levels in chives, and both red and white garlic were in agreement with the enriched content of tetrathiaoctane (peak 128) and 2,4,5-tetrathiahexane (peak 74) and diallyldisulfide (peak 60), also explaining its segregation on the right side of the loading plot. Moreover, the highest propyl disulfide level (peak 69) along with fatty acids mainly palmitic acid (peak 148) in red and white onion and French and Egyptian leek accounted for their segregation on the left side of the score plot. The segregation of French and Egyptian leek together is opposite to that observed in nutrient modeling, and suggestive that aroma provides a better model for Allium classification.

### 3.6. Unsupervised PCA Model Using Aroma Sulfur Compounds

**Figure 5:**
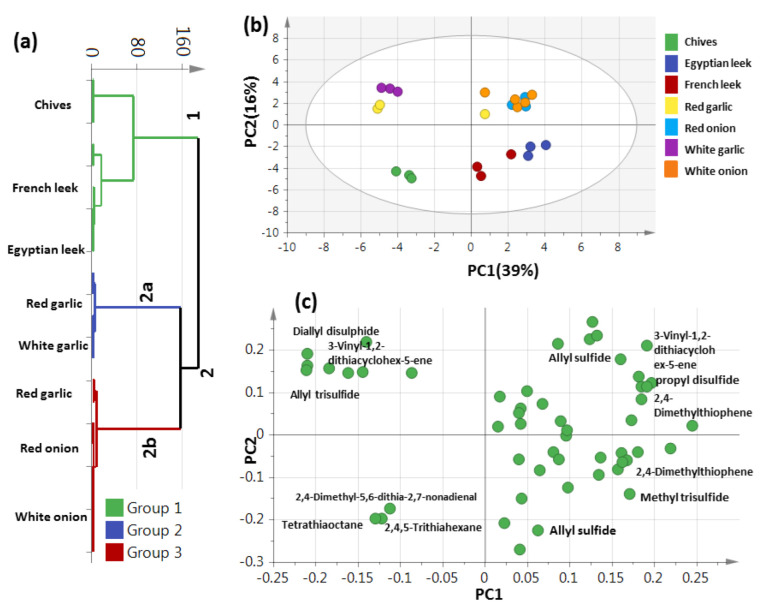
Unsupervised multivariate data analyses of the studied Allium species derived from the modeling of sulfur metabolites analyzed via GC-MS (n = 3). (**a**) HCA plot. (**b**) PCA score plot of PC1 vs. PC2 scores. (**c**) The respective loading plot for PC1 and PC2, providing mass peaks and their assignments. The metabolome clusters are placed in two-dimensional space at the distinct locations defined by two vectors of principal component PC1 = 39% and PC2 = 16%.

OSCs as the major metabolites volatile class in Allium species amounting to 29.2–86.6% of total aroma were used to assess variation among different samples using HCA and PCA models (Figure 5). HCA depicted a dendrogram of three distinct clusters (Figure 5a), where chives and Egyptian and French leek were clustered in group 1, red and white garlic were clustered in 2a, and red garlic and red and white onion were positioned into two subdivisions from group 2b. A PCA model (Figure 5b), consisting of two orthogonal PCs that accounted for 55% of the total variance, showed clear discrimination of chives on the negative left side and red and white garlic clusters on the positive left side of PC1. In contrast, towards the right side of PC1 were two clusters; one for Egyptian and French leek at the lower right side and the other for red and white onion with red garlic at the upper side, separable along PC2. The PCA plot revealed discrimination of the edible Allium from the inedible aerial parts depending on variation in sulfur metabolites and suggestive that Sulphur compounds provide the best model for such categorization of edible versus inedible. The corresponding loading plot (Figure 5c) revealed that the higher OSCs levels were detected in chives in agreement with the enriched content of tetrathiaoctane (peak 128) and 2,4,5-tetrathiahexane (peak 74), explaining its segregation on the lower left side of the loading plot. Red and white garlic were enriched with diallyl disulfide (peak 60) and allyl trisulfide (peak 107), explaining their segregation at the upper left side of the loading plot. Moreover, the highest content in propyl disulfide (peak 69) and Allyl sulfide (peak 8) in French and Egyptian leek accounted for their segregation in the lower right side of the loading plot. Additionally, propyl disulfide (peak 69) and allyl sulfide (peak 111) as the marker metabolites in red and white onion, helped their segregation in the upper right side. 

### 3.7. Supervised OPLS-DA Analysis of Allium Rich in Sulfur versus Those Rich in Other Volatile Classes 

**Figure 6:**
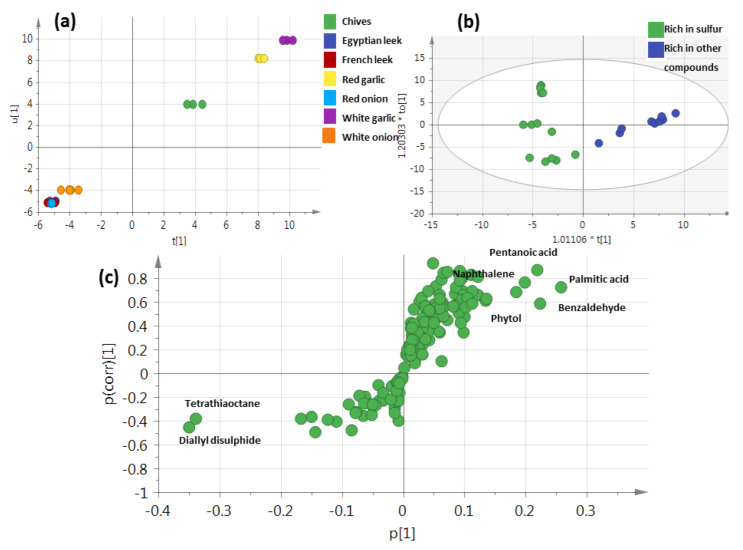
GC-MS-based OPLS-DA score plot (**a**) derived from modeling volatile metabolites of plants rich in sulphur compounds versus plants rich in other compounds (n = 3). (**b**) OPLS French leek, Egyptian leek, and red onion that clustered at left in one class against the other species that clustered at right. (**c**) The respective loading S-plots showing the covariance p [1] against the correlation p [6] [1] of the variables of the discriminating component of the OPLS-DA model are depicted in (**c**). Cut-off values of *p* < 9.09758 × 10^−4^ were used. Designated variables are highlighted and identifications are discussed in the text.

The volatile profile of Allium species aerial parts using a supervised OPLS-DA model (Figure 6) was further employed to assess their variation by constructing a model to assess the inner relationship between Allium species. Figure 6a revealed the segregation of inedible Allium, viz., chives, and red and white garlic on the upper right side and the edible Allium, viz., French, Egyptian leek, and red and white onion on the lower left side. Other OPLS-DA models of plants rich in sulfur against those rich in other metabolites (Figure 6b) showed Q2 = 0.87 and 0.61 and R2 = 0.88 and 0.71 and a p-value of less than 0.05, indicating the predictability of the model. The respective loading S-plot (Figure 6c) revealed that sulfur compounds are rich in chives and red and white garlic, represented by tetrathiaoctane (peak 128) and diallyl disulfide (peak 60). 

## 4. Conclusions

Metabolite heterogeneity in both nutrient and volatile profiles of edible and inedible aerial parts from seven Allium species is introduced herein for the first time through holistic untargeted MS-based metabolomics. A total of 77 nutrient metabolites were detected, including mostly sugars (mono- and disaccharides), sugar alcohols, sugar acids, fatty acids/esters, organic acids, amino acids, nitrogenous compounds, and alcohols. Glucose, fructose, and mannose were detected as the most abundant monosaccharides in edible Alliums. Sucrose was detected in all Allium aerial parts. Inedible white garlic leafy parts contained 20% sugar compared to other inedible taxa. Multivariate data analysis of nutrients revealed that leeks were the most distinct among Alliums with the highest sugar, fatty acid/esters, and amino acids, respectively. Linoleic acid and α-linolenic acid are the most important PUFA detected in Allium species. A total of 148 volatiles were identified in Allium aerial parts accounting for their aroma with the abundance of sulfur, aldehydes/ketones, and alcohols, in all Allium aerial parts. Chive aerial parts contained the highest levels of sulfur compounds, being abundant in tetrathiaoctane as a marker metabolite. French leek was different in the nutrients profile and rich in octadien-2-one, posing it as the freshest and most palatable among leafy Allium. Our study provided the first complementary phytochemical evidence that supports the nutritional and flavor determinants of edible Allium aerial parts to be distinguished from inedible ones to corroborate their utilization as functional foods or for further incorporation in nutraceuticals.

## Figures and Tables

**Table 1:** Quantification of silylated metabolites in Allium species aerial part (mg/g) analyzed using GC–MS, *n* = 3.

Peak Number	Average Rt	Average RI	Metabolite	Class	Chives	Egyptian Leek	French Leek	Red Garlic	Red Onion	White Garlic	White Onion
3	5.13	1061.59	Lactic acid, 2TMS	Acid	0.24 ± 0.12	0.23 ± 0.03	0.40 ± 0.12	0.30 ± 0.11	0.26 ± 0.18	0.29 ± 0.11	0.37 ± 0.12
4	5.34	1074.61	Glycolic acid, 2TMS	Acid	0.05 ± 0.01	0.06 ± 0.01	0.17 ± 0.02	0.05 ± 0.00	0.05 ± 0.01	0.05 ± 0.01	0.05 ± 0.00
5	5.528	1086.41	Pyruvic acid, 2TMS	Acid	0.05 ± 0.02	0.19 ± 0.07	0.34 ± 0.02	0.10 ± 0.02	0.14 ± 0.04	0.03 ± 0.01	0.09 ± 0.03
6	5.649	1093.85	Octanoic acid, TMS	Acid	0.02 ± 0.01	0.02 ± 0.00	0.02 ± 0.00	0.02 ± 0.00	0.02 ± 0.00	0.02 ± 0.00	0.02 ± 0.00
8	6.365	1138.27	β-Lactic acid, 2TMS	Acid	0.02 ± 0.00	0.03 ± 0.01	0.16 ± 0.02	0.02 ± 0.00	0.02 ± 0.00	0.02 ± 0.00	0.03 ± 0.00
11	7.704	1224.87	γ-Hydroxybutyric acid, 2TMS	Acid	0.36 ± 0.08	0.40 ± 0.05	0.48 ± 0.01	0.41 ± 0.04	0.38 ± 0.07	0.40 ± 0.05	0.42 ± 0.06
14	8.23	1262.65	Methylmaleic acid, 2TMS	Acid	0.03 ± 0.01	0.08 ± 0.06	0.09 ± 0.09	0.04 ± 0.00	0.03 ± 0.00	0.03 ± 0.00	0.04 ± 0.01
17	8.808	1304.12	Succinic acid, 2TMS	Acid	0.17 ± 0.04	0.32 ± 0.06	1.23 ± 0.10	0.17 ± 0.01	0.29 ± 0.05	0.18 ± 0.03	0.24 ± 0.06
20	9.248	1335.82	Fumaric acid, 2TMS	Acid	0.02 ± 0.00	0.03 ± 0.00	0.24 ± 0.07	0.01 ± 0.00	0.06 ± 0.01	0.00 ± 0.00	0.07 ± 0.02
24	10.057	1393.63	Methylsuccinic acid, 2TMS	Acid	0.00 ± 0.00	0.00 ± 0.00	0.02 ± 0.00	0.00 ± 0.00	0.00 ± 0.00	0.00 ± 0.00	0.00 ± 0.00
27	10.65	1440.33	Itaconic acid, 2TMS	Acid	0.05 ± 0.02	0.00 ± 0.00	0.03 ± 0.01	0.00 ± 0.00	0.02 ± 0.01	0.00 ± 0.00	0.01 ± 0.01
30	10.952	1465.15	2-Ketoisocaproic acid tbo, TMS	Acid	0.20 ± 0.06	0.22 ± 0.05	0.27 ± 0.02	0.25 ± 0.04	0.21 ± 0.05	0.25 ± 0.06	0.21 ± 0.05
31	11.013	1469.98	Succinic acid, 2TMS	Acid	0.00 ± 0.00	0.03 ± 0.01	0.03 ± 0.01	0.00 ± 0.00	0.01 ± 0.00	0.00 ± 0.00	0.02 ± 0.01
32	11.205	1485.5	Malic acid, 3TMS	Acid	3.13 ± 0.97	0.39 ± 0.09	3.58 ± 0.32	0.32 ± 0.12	2.28 ± 0.40	0.16 ± 0.09	0.92 ± 0.23
50	13.885	1712.97	2-Ketoglutaric acid, 3TMS	Acid	0.02 ± 0.01	0.00 ± 0.00	0.00 ± 0.00	0.00 ± 0.00	0.01 ± 0.01	0.00 ± 0.00	0.01 ± 0.01
56	14.644	1781.21	Azelaic acid, 2TMS	Acid	0.00 ± 0.00	0.01 ± 0.00	0.01 ± 0.00	0.00 ± 0.00	0.01 ± 0.00	0.01 ± 0.00	0.01 ± 0.00
Total Acid					4.36 ± 1.35	2.02 ± 0.45	7.08 ± 0.83	1.70 ± 0.35	3.79 ± 0.83	1.46 ± 0.37	2.51 ± 0.59
2	5.048	1056.5	1,3 Propanediol, 2TMS	Alcohol	0.03 ± 0.01	0.03 ± 0.00	0.03 ± 0.00	0.03 ± 0.00	0.03 ± 0.00	0.03 ± 0.00	0.03 ± 0.00
12	7.841	1234.73	Diethylene glycol, 2TMS	Alcohol	0.05 ± 0.01	0.05 ± 0.00	0.05 ± 0.00	0.05 ± 0.00	0.05 ± 0.01	0.05 ± 0.00	0.05 ± 0.00
25	10.073	1394.89	1-Nonanol, TMS	Alcohol	0.01 ± 0.00	0.01 ± 0.00	0.01 ± 0.00	0.01 ± 0.00	0.01 ± 0.00	0.01 ± 0.00	0.01 ± 0.00
Total Alcohol					0.08 ± 0.02	0.09 ± 0.01	0.09 ± 0.00	0.09 ± 0.01	0.09 ± 0.01	0.10 ± 0.01	0.09 ± 0.01
7	5.758	1100.63	l-Alanine, TMS	Amino acid	0.22 ± 0.15	2.62 ± 0.61	1.89 ± 0.49	0.07 ± 0.04	0.85 ± 0.08	0.31 ± 0.10	2.36 ± 2.12
9	7.389	1202.33	Glycine, TMS	Amino acid	0.48 ± 0.12	0.50 ± 0.07	0.52 ± 0.03	0.53 ± 0.05	0.51 ± 0.04	0.50 ± 0.05	0.50 ± 0.03
10	7.464	1207.63	Valine, 2TMS	Amino acid	0.05 ± 0.02	3.51 ± 1.09	1.75 ± 0.51	0.03 ± 0.01	0.22 ± 0.04	0.32 ± 0.14	1.00 ± 0.49
15	8.276	1265.93	L-Leucine, TMS	Amino acid	0.02 ± 0.01	5.32 ± 1.44	3.22 ± 0.92	0.02 ± 0.01	0.20 ± 0.03	0.47 ± 0.22	1.41 ± 0.61
16	8.588	1288.31	L-Norleucine, TMS	Amino acid	0.02 ± 0.01	2.34 ± 0.75	1.00 ± 0.29	0.01 ± 0.01	0.08 ± 0.01	0.22 ± 0.12	0.61 ± 0.30
21	9.54	1356.67	Serine, 3TMS	Amino acid	0.06 ± 0.02	1.34 ± 0.38	0.45 ± 0.19	0.04 ± 0.02	0.40 ± 0.07	0.20 ± 0.08	0.91 ± 0.28
22	9.911	1383.42	L-Threonine, 3TMS	Amino acid	0.04 ± 0.01	1.10 ± 0.32	0.47 ± 0.14	0.03 ± 0.01	0.11 ± 0.03	0.19 ± 0.07	0.38 ± 0.11
26	10.376	1418.66	β-Alanine, 2TMS	Amino acid	0.11 ± 0.03	0.11 ± 0.02	0.12 ± 0.01	0.13 ± 0.02	0.11 ± 0.02	0.11 ± 0.02	0.11 ± 0.01
28	10.742	1448.19	Leucine, 2TBDMS	Amino acid	0.00 ± 0.00	0.00 ± 0.00	0.00 ± 0.00	0.00 ± 0.00	0.00 ± 0.00	0.00 ± 0.00	0.00 ± 0.00
29	10.918	1462.37	L-Aspartic acid, 3TMS	Amino acid	0.22 ± 0.11	0.07 ± 0.03	0.08 ± 0.02	0.00 ± 0.00	0.02 ± 0.01	0.01 ± 0.00	0.05 ± 0.02
35	11.583	1516.01	Pyroglutamic acid, 2TMS	Amino acid	0.10 ± 0.01	0.88 ± 0.32	4.10 ± 0.91	0.05 ± 0.02	0.19 ± 0.03	0.07 ± 0.01	0.34 ± 0.12
36	11.591	1516.99	Aspartic acid, 3TMS	Amino acid	0.05 ± 0.02	0.35 ± 0.10	0.10 ± 0.09	0.01 ± 0.01	0.16 ± 0.03	0.01 ± 0.00	0.11 ± 0.03
43	12.767	1612.66	Glutamic acid, 3TMS	Amino acid	0.17 ± 0.07	1.71 ± 0.54	0.24 ± 0.05	0.07 ± 0.02	0.44 ± 0.12	0.07 ± 0.02	0.38 ± 0.15
44	12.843	1619.48	Phenylalanine, 2TMS	Amino acid	0.02 ± 0.01	2.04 ± 0.66	0.85 ± 0.22	0.01 ± 0.01	0.10 ± 0.03	0.18 ± 0.08	0.61 ± 0.24
45	12.951	1629.1	Asparagine, 3TMS	Amino acid	0.02 ± 0.02	0.04 ± 0.02	0.04 ± 0.02	0.00 ± 0.00	0.09 ± 0.01	0.03 ± 0.01	0.06 ± 0.03
48	13.368	1666.69	L-Asparagine, 3TMS	Amino acid	0.07 ± 0.04	0.16 ± 0.06	0.10 ± 0.05	0.00 ± 0.00	0.22 ± 0.06	0.07 ± 0.04	0.15 ± 0.08
52	14.112	1733.57	L-Glutamine, 2TMS	Amino acid	0.03 ± 0.02	0.23 ± 0.08	0.11 ± 0.02	0.01 ± 0.00	0.20 ± 0.02	0.03 ± 0.01	0.16 ± 0.12
54	14.48	1766.68	L-Glutamine, 2TMS	Amino acid	0.16 ± 0.07	1.48 ± 0.61	0.90 ± 0.20	0.06 ± 0.02	1.18 ± 0.35	0.37 ± 0.12	0.93 ± 0.51
65	16.209	1934.73	Tyrosine, 3TMS	Amino acid	0.02 ± 0.00	1.33 ± 0.42	1.00 ± 0.34	0.02 ± 0.01	0.08 ± 0.02	0.12 ± 0.06	0.43 ± 0.21
Total Amino Acid					1.86 ± 0.74	25.13 ± 7.51	16.94 ± 4.51	1.10 ± 0.25	5.16 ± 1.01	3.27 ± 1.15	10.50 ± 5.46
33	11.403	1501.26	Methyl octanoate, TMS	Ester	0.14 ± 0.03	0.13 ± 0.02	0.15 ± 0.02	0.13 ± 0.01	0.14 ± 0.02	0.13 ± 0.01	0.13 ± 0.01
Total Ester					0.14 ± 0.03	0.13 ± 0.02	0.15 ± 0.02	0.13 ± 0.01	0.14 ± 0.02	0.13 ± 0.01	0.13 ± 0.01
77	23.124	2759.61	Monostearin, 2TMS	Fatty acid /Ester	0.35 ± 0.24	0.23 ± 0.24	6.71 ± 1.54	0.37 ± 0.52	0.54 ± 0.25	0.75 ± 0.71	0.37 ± 0.28
68	17.071	2022.13	Palmitic acid, TMS	Fatty acid/Ester	1.80 ± 0.46	2.45 ± 0.38	1.99 ± 0.04	1.75 ± 0.77	1.69 ± 0.18	2.30 ± 0.62	1.91 ± 0.61
71	18.593	2187.5	Linoleic acid, TMS	Fatty acid/Ester	0.09 ± 0.02	0.15 ± 0.11	0.12 ± 0.05	0.05 ± 0.03	0.06 ± 0.02	0.12 ± 0.03	0.07 ± 0.03
72	18.636	2192.26	Oleic acid, TMS	Fatty acid/Ester	0.09 ± 0.02	0.06 ± 0.02	0.08 ± 0.02	0.05 ± 0.02	0.06 ± 0.02	0.06 ± 0.02	0.07 ± 0.01
73	18.656	2194.49	α-Linolenic acid, TMS	Fatty acid/Ester	0.09 ± 0.02	0.42 ± 0.09	0.41 ± 0.06	0.06 ± 0.02	0.07 ± 0.02	0.12 ± 0.03	0.12 ± 0.02
74	18.85	2216.87	Stearic acid, TMS	Fatty acid/Ester	2.04 ± 0.66	2.23 ± 0.26	2.53 ± 0.03	2.11 ± 0.46	1.91 ± 0.13	2.11 ± 0.37	2.03 ± 0.40
75	21.717	2568.34	1-Monopalmitin TMS	Fatty acid/Ester	0.63 ± 0.43	0.43 ± 0.45	7.32 ± 1.68	0.64 ± 0.97	0.97 ± 0.51	1.40 ± 1.30	0.68 ± 0.52
Total Fatty Acid/Ester					5.08 ± 1.85	5.98 ± 1.55	19.15 ± 3.43	5.02 ± 2.78	5.30 ± 1.13	6.87 ± 3.07	5.25 ± 1.86
13	8.181	1259.15	Nicotinic acid-TMS	Nitrogenous	0.03 ± 0.01	0.03 ± 0.00	0.05 ± 0.00	0.04 ± 0.00	0.03 ± 0.00	0.03 ± 0.01	0.03 ± 0.00
19	9.202	1332.41	Uracil, 2TMS	Nitrogenous	0.00 ± 0.00	0.03 ± 0.01	0.02 ± 0.00	0.00 ± 0.00	0.02 ± 0.00	0.00 ± 0.00	0.05 ± 0.02
23	9.981	1388.3	Ethanolamine, 2TMS	Nitrogenous	0.74 ± 0.17	0.71 ± 0.07	0.69 ± 0.04	0.73 ± 0.04	0.76 ± 0.02	0.75 ± 0.03	0.70 ± 0.05
37	11.677	1523.47	γ-Aminobutyric acid, 3TMS	Nitrogenous	0.02 ± 0.01	1.13 ± 0.48	0.88 ± 0.33	0.01 ± 0.00	0.25 ± 0.10	0.17 ± 0.08	0.64 ± 0.26
Total Nitrogenous					0.79 ± 0.20	1.90 ± 0.56	1.64 ± 0.37	0.78 ± 0.04	1.06 ± 0.13	0.96 ± 0.12	1.41 ± 0.33
1	4.842	1043.72	L-Threose, 3TMS (isomer 1)	Sugar	0.04 ± 0.01	0.04 ± 0.00	0.04 ± 0.00	0.04 ± 0.00	0.04 ± 0.01	0.04 ± 0.00	0.04 ± 0.00
41	12.459	1586.48	D-Psicose, 5TMS	Sugar	0.46 ± 0.21	0.28 ± 0.11	1.57 ± 0.27	0.15 ± 0.09	0.04 ± 0.01	0.26 ± 0.12	0.18 ± 0.04
42	12.58	1596.35	D-Tagatofuranose, 5TMS (isomer 2)	Sugar	0.00 ± 0.00	0.01 ± 0.01	0.01 ± 0.00	0.00 ± 0.00	0.01 ± 0.00	0.01 ± 0.00	0.02 ± 0.00
46	12.985	1632.41	L-Rhamnopyranose, 4TMS	Sugar	0.01 ± 0.00	0.01 ± 0.00	0.02 ± 0.00	0.00 ± 0.00	0.00 ± 0.00	0.00 ± 0.00	0.00 ± 0.00
47	13.225	1654.44	Arabinose, 4TMS	Sugar	0.00 ± 0.00	0.02 ± 0.01	0.01 ± 0.00	0.00 ± 0.00	0.00 ± 0.00	0.00 ± 0.00	0.00 ± 0.00
49	13.418	1671.58	Arabinose, 4TMS	Sugar	0.00 ± 0.00	0.02 ± 0.01	0.01 ± 0.00	0.00 ± 0.00	0.01 ± 0.00	0.01 ± 0.01	0.02 ± 0.01
51	13.899	1714.3	α-D-Lyxopyranose, 4TMS	Sugar	0.01 ± 0.00	0.01 ± 0.00	0.02 ± 0.00	0.00 ± 0.00	0.02 ± 0.01	0.05 ± 0.01	0.03 ± 0.01
57	14.984	1813.12	D-Tagatofuranose, 5TMS (isomer 1)	Sugar	0.16 ± 0.09	0.57 ± 0.17	3.77 ± 0.23	0.12 ± 0.06	0.96 ± 0.17	0.56 ± 0.39	1.96 ± 0.46
58	15.066	1821.23	D-Tagatofuranose, 5TMS(isomer 2)	Sugar	0.57 ± 0.24	1.68 ± 0.45	11.37 ± 0.79	0.43 ± 0.16	3.29 ± 0.77	2.01 ± 1.15	5.96 ± 1.08
59	15.145	1829.16	D-Fructopyranose, 5TMS (isomer 1)	Sugar	1.39 ± 0.47	3.86 ± 1.74	16.29 ± 1.28	0.66 ± 0.32	6.46 ± 1.50	4.36 ± 2.26	10.70 ± 1.54
60	15.383	1852.71	D-Talofuranose, 5TMS (isomer 2)	Sugar	0.05 ± 0.03	0.06 ± 0.03	0.26 ± 0.04	0.03 ± 0.03	0.11 ± 0.01	0.05 ± 0.03	0.19 ± 0.04
61	15.48	1862.34	D-(-)-Fructopyranose, 5TMS (isomer 1)	Sugar	0.02 ± 0.01	0.09 ± 0.04	0.29 ± 0.03	0.01 ± 0.01	0.11 ± 0.02	0.06 ± 0.04	0.22 ± 0.04
62	15.854	1899.54	D-Psicose, 5TMS	Sugar	0.06 ± 0.03	0.36 ± 0.16	1.47 ± 0.14	0.03 ± 0.02	0.50 ± 0.11	0.24 ± 0.21	0.95 ± 0.21
63	15.892	1903.29	β-D-Mannopyranose, 5TMS	Sugar	2.82 ± 1.02	3.34 ± 1.15	12.70 ± 1.81	0.37 ± 0.15	8.68 ± 1.73	3.92 ± 2.63	13.67 ± 1.91
64	16.006	1914.56	D-Glucose, 5TMS	Sugar	0.03 ± 0.01	0.54 ± 0.11	0.51 ± 0.05	0.02 ± 0.01	0.06 ± 0.01	0.12 ± 0.05	0.31 ± 0.08
67	16.747	1988.06	β-D-Glucopyranose, 5TMS	Sugar	3.95 ± 1.31	4.68 ± 1.56	15.88 ± 2.09	0.49 ± 0.21	11.22 ± 2.13	5.32 ± 3.48	17.07 ± 2.24
76	22.495	2673.08	Sucrose, 8TMS	Sugar	2.29 ± 0.67	0.19 ± 0.12	10.34 ± 2.54	1.45 ± 0.63	3.47 ± 0.91	3.03 ± 1.90	1.93 ± 1.97
Total Sugar					11.87 ± 4.10	15.76 ± 5.70	74.55 ± 9.29	3.80 ± 1.68	34.97 ± 7.40	20.04 ± 12.29	53.25 ± 9.62
18	9.142	1328.08	Glyceric acid, 3TMS	Sugar acid	0.24 ± 0.07	1.35 ± 0.59	1.71 ± 0.26	0.84 ± 0.09	0.17 ± 0.05	0.18 ± 0.06	0.24 ± 0.05
39	11.972	1547.35	Erythronic acid, 4TMS	Sugar acid	0.01 ± 0.00	0.01 ± 0.00	0.04 ± 0.01	0.01 ± 0.00	0.01 ± 0.00	0.01 ± 0.00	0.01 ± 0.01
40	12.172	1563.47	L-Threonic acid, 4TMS	Sugar acid	0.01 ± 0.00	0.02 ± 0.00	0.08 ± 0.01	0.06 ± 0.02	0.44 ± 0.11	0.05 ± 0.01	0.44 ± 0.12
53	14.437	1762.66	Glucaric acid, 6TMS	Sugar acid	0.00 ± 0.00	0.00 ± 0.00	0.01 ± 0.00	0.00 ± 0.00	0.00 ± 0.00	0.00 ± 0.00	0.00 ± 0.00
55	14.583	1775.59	Ribonic acid, 5TMS	Sugar acid	0.00 ± 0.00	0.01 ± 0.00	0.00 ± 0.00	0.00 ± 0.00	0.00 ± 0.00	0.00 ± 0.00	0.00 ± 0.00
69	17.156	2031.28	D-Glucuronic acid, 5TMS	Sugar acid	0.00 ± 0.00	0.01 ± 0.00	0.01 ± 0.00	0.01 ± 0.00	0.01 ± 0.00	0.01 ± 0.00	0.01 ± 0.00
Total Sugar Acid					0.26 ± 0.08	1.39 ± 0.60	1.85 ± 0.27	0.91 ± 0.11	0.64 ± 0.16	0.25 ± 0.08	0.71 ± 0.19
34	11.526	1511.17	Erythritol, 4TMS	Sugar alcohol	0.00 ± 0.00	0.01 ± 0.00	0.03 ± 0.01	0.00 ± 0.00	0.01 ± 0.00	0.01 ± 0.00	0.01 ± 0.01
38	11.931	1543.65	Threitol, 4TMS	Sugar alcohol	0.00 ± 0.00	0.00 ± 0.00	0.01 ± 0.01	0.00 ± 0.00	0.01 ± 0.00	0.00 ± 0.00	0.02 ± 0.02
66	16.384	1952.03	Mannitol, 6TMS	Sugar alcohol	0.00 ± 0.00	0.01 ± 0.00	0.05 ± 0.05	0.01 ± 0.00	0.08 ± 0.02	0.01 ± 0.01	0.13 ± 0.15
70	17.825	2104.04	Myoinositol, 6TMS	Sugar alcohol	0.24 ± 0.07	0.17 ± 0.04	0.68 ± 0.07	0.07 ± 0.03	0.15 ± 0.04	0.06 ± 0.02	0.28 ± 0.05
Total Sugar Alcohol					0.24 ± 0.07	0.18 ± 0.05	0.77 ± 0.14	0.08 ± 0.03	0.25 ± 0.06	0.08 ± 0.03	0.44 ± 0.23

## Data Availability

Data are available within the article.

## Supplementary Materials

The following supporting information can be downloaded at: https://www.mdpi.com/article/10.3390/metabo13010018/s1, Figure S1: GC–MS-based OPLS-DA score plot (**a**) derived from modeling silylated primary metabolites of white onion versus the other 6 *Allium* species (n = 3). (**b**) Derived from modeling silylated primary metabolites of French leek versus other 6 *Allium* species (n = 3). (**c**) Derived from modeling silylated primary metabolites of Egyptian leek versus other 6 *Allium* species (n = 3). The respective loading S-plots showing the covariance p [1] against the correlation p(cor) [1] of the variables of the discriminating component of the OPLS-DA model are depicted in (**d**), (**e**), and (**f**). Cut-off values of *p* < 1.61967 × 10^−8^, 1.78505 × 10^−11^ and 0.000217456 were used. Designated variables are highlighted and identifications are discussed in the text. Figure S2: Representative GC–MS chromatograms of volatile constituents of 7 different *Allium* species. **37**, 2,4-Dimethylthiazole; **41**, Pseudocumene; **60**, Diallyl disulphide; **69**, Propyl disulfide, **74**, 2,4,5-Trithiahexane; **128**, Tetrathiaoctane; **148**, Palmitic acid. Figure S3: Different volatile classes identified in seven *Allium* species. Figure S4: Pie chart illustrating distribution of different volatile classes identified in seven *Allium* species. Table S1: Relative percentage of volatile metabolites in *Allium* species aerial parts analyzed via GC–MS, n = 3.

## Recommendations

Authors recommend the following for continuing studies on this topic: (1) an extended approach utilizing liquid chromatography coupled with mass spectrometry (LC-MS) to pinpoint differences in bioactive secondary metabolite profiles among Allium aerial parts accessions likely to contribute to health benefits. (2) Assessing the nutritional impact of studied Allium species on animals and humans, etc., as a next step based on chemical findings. Screening the antidiabetic potential of Allium species owing to the detection of sugar alcohols is recommended among accessions enriched in that class. (3) Determination of cardiovascular and antihyperlipidemic assays for inedible *Allium*, viz., chives, white garlic, and red garlic found to be most enriched in sulphur compounds.
