# Supplementary material for: How Does Allium Leafy Parts Metabolome Differ in Context to Edible or Inedible Taxa? Case Study in Seven Allium Species as Analyzed Using MS-Based Metabolomics
Source: Metabolites. 2022 Dec 22;13(1):18. doi: 10.3390/metabo13010018 (PMC9866920; doi:10.3390/metabo13010018)
Supplement: Supplementary file 1 [file metabolites-13-00018-s001.zip › metabolites-2096331-supplementary.pdf]

Supplementary Materials for *Metabolites*

How does Allium leafy parts metabolome differ in context to edible or not edible taxa? Case study in 7 Allium species as analyzed using MS based metabolomics

Mostafa H. Baky[1], Samir Shamma[2], Mohamed R. Khalifa[2], Mohamed A. Farag[3*]

[1] *Department of Pharmacognosy, Faculty of pharmacy, Egyptian Russian University, Badr city, 11829, Cairo, Egypt.*

[2]*Institute of Global Health and Human Ecology, School of Sciences and Engineering, The American University in Cairo, P.O. Box 74, New Cairo 11835, Egypt*

[3] *Pharmacognosy Department, College of Pharmacy, Cairo University, 11562 Cairo, Egypt.*

*Corresponding author at: Cairo University, College of Pharmacy, Department of Pharmacognosy, Egypt.

E-mail addresses: mohamed.farag@pharma.cu.edu.eg, (M.A. Farag).

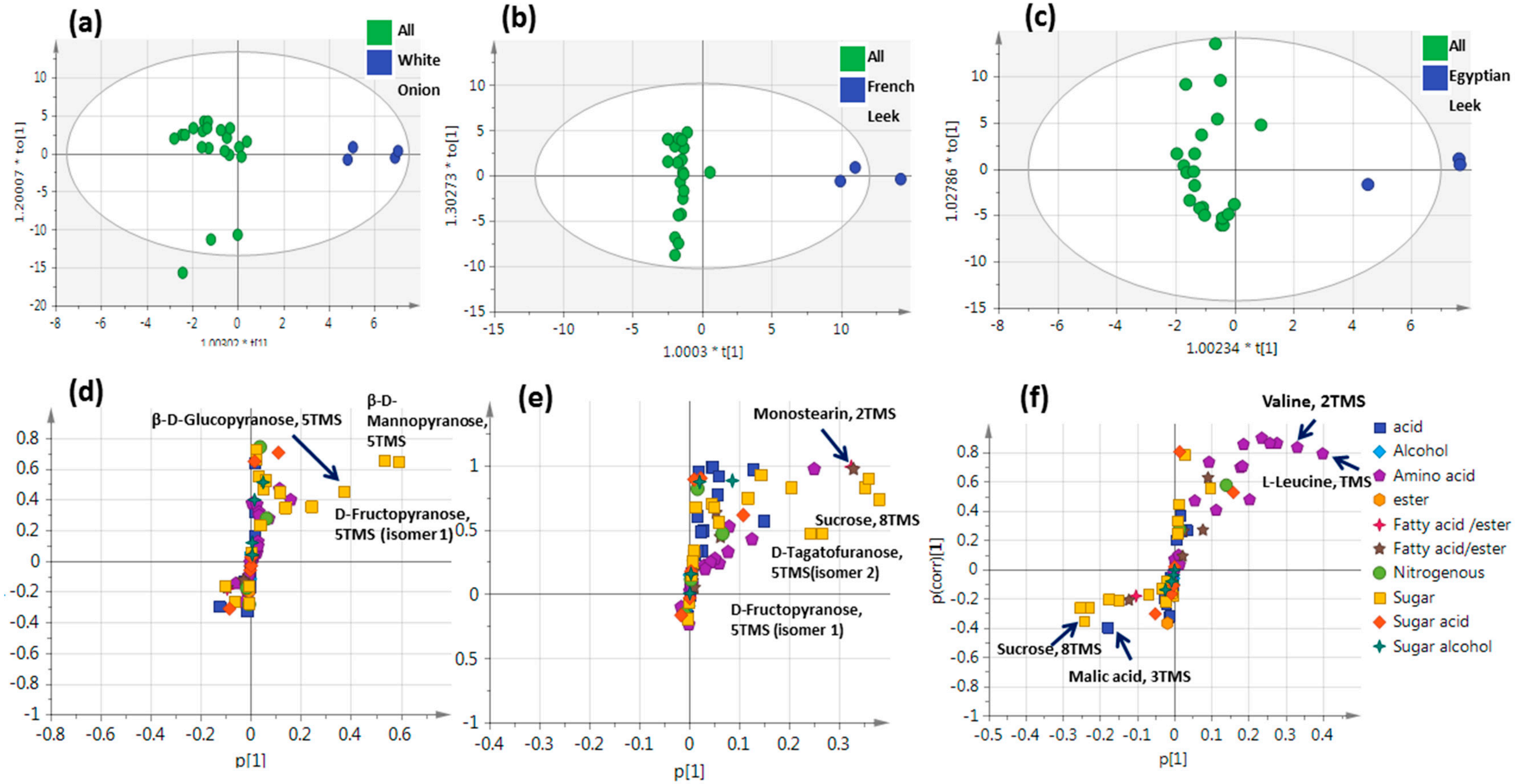

(a)
All
White
Onion
1.20007 * to[1]
1.00302 * t[1]
(b)
All
French
Leek
1.30273 * to[1]
1.0003 * t[1]
(c)
All
Egyptian
Leek
1.02786 * to[1]
1.00234 * t[1]
(d)
β-D-Glucopyranose, 5TMS
β-D-Mannopyranose, 5TMS
D-Fructopyranose, 5TMS (isomer 1)
p[1]
(e)
Monostearin, 2TMS
Sucrose, 8TMS
D-Tagatofuranose, 5TMS(isomer 2)
D-Fructopyranose, 5TMS (isomer 1)
p[1]
(f)
Valine, 2TMS
L-Leucine, TMS
Sucrose, 8TMS
Malic acid, 3TMS
p(corr)[1]
p[1]
acid
Alcohol
Amino acid
ester
Fatty acid /ester
Fatty acid/ester
Nitrogenous
Sugar
Sugar acid
Sugar alcohol

Figure S1. GC–MS-based OPLS-DA score plot (**a**) derived from modeling silylated primary metabolites of white onion versus the other 6 *Allium* species (n = 3). (**b**) Derived from modeling silylated primary metabolites of French leek versus other 6 *Allium* species (n = 3). (**c**) Derived from modeling silylated primary metabolites of Egyptian leek versus other 6 *Allium* species (n = 3). The respective loading S-plots showing the covariance p [1] against the correlation p(cor) [1] of the variables of the discriminating component of the OPLS-DA model are depicted in (**d**), (**e**), and (**f**). Cut-off values of $p < 1.61967e-008$, 1.78505e-011 and 0.000217456 were used. Designated variables are highlighted and identifications are discussed in the text.

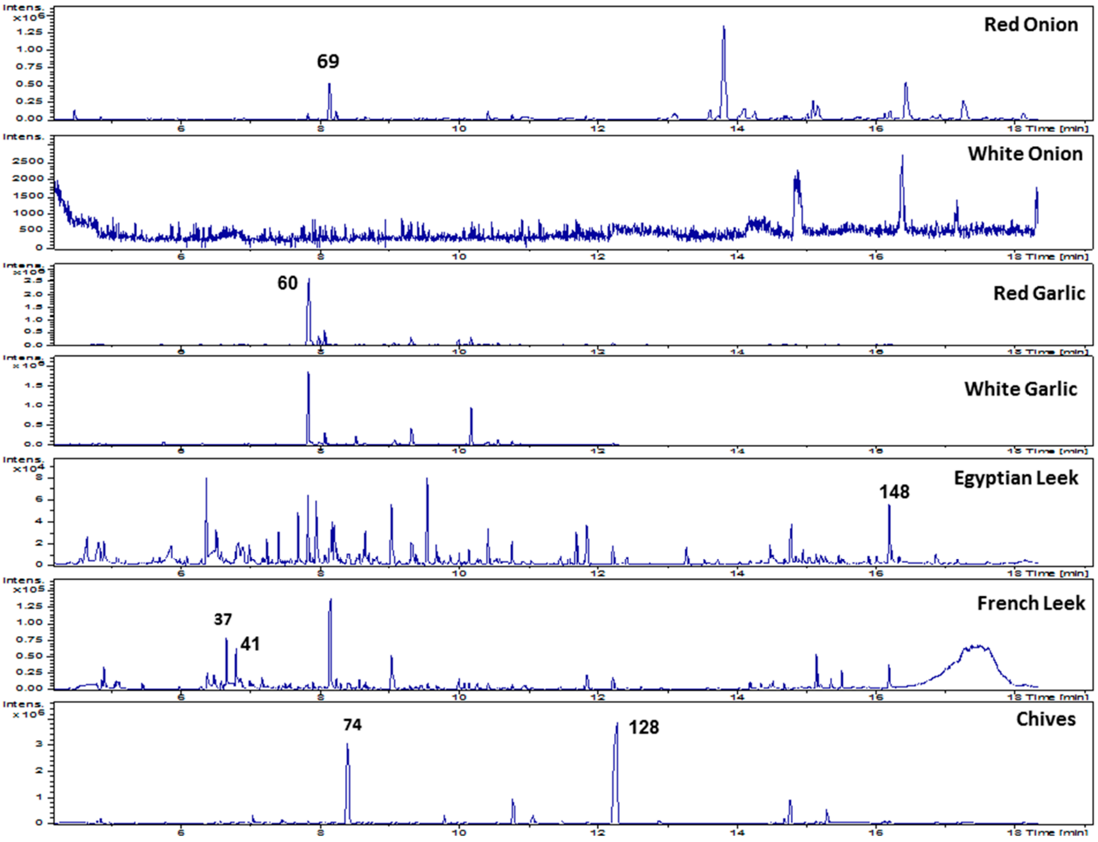

Figure S2. Representative GC–MS chromatograms of volatile constituents of 7 different *Allium* species. **37**, 2,4-Dimethylthiazole**; 41,** Pseudocumene**; 60,** Diallyl disulphide**; 69,** Propyl disulfide, **74**, 2,4,5-Trithiahexane**; 128,** Tetrathiaoctane**; 148,** Palmitic acid

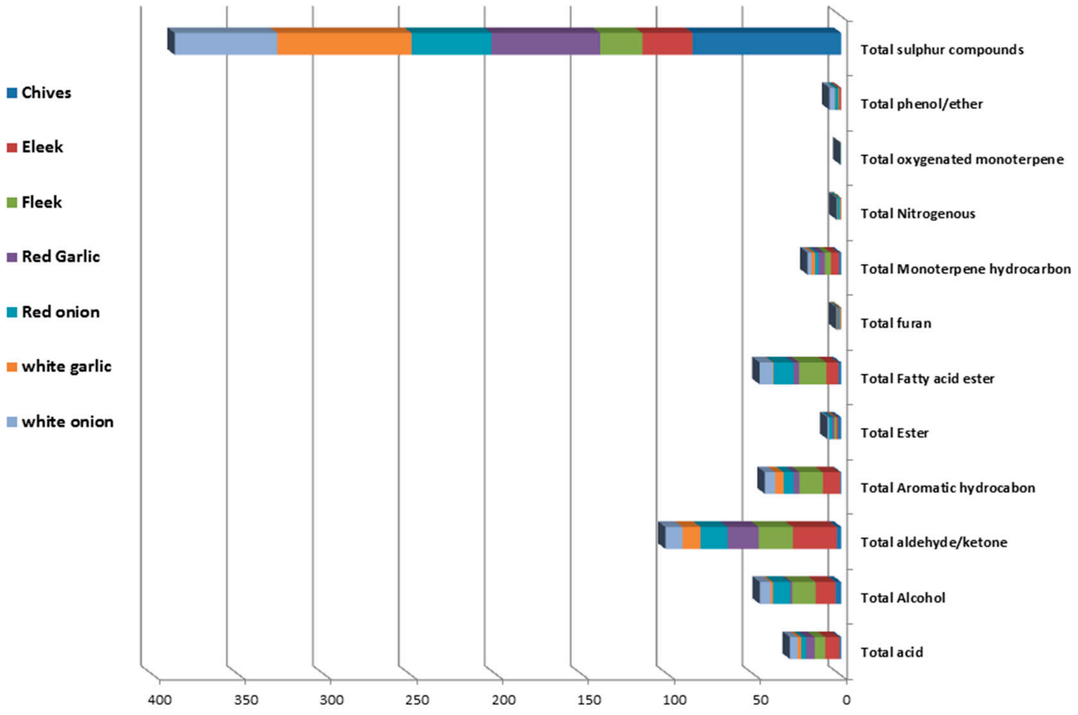

Figure S3. Different volatile classes identified in seven *Allium* species

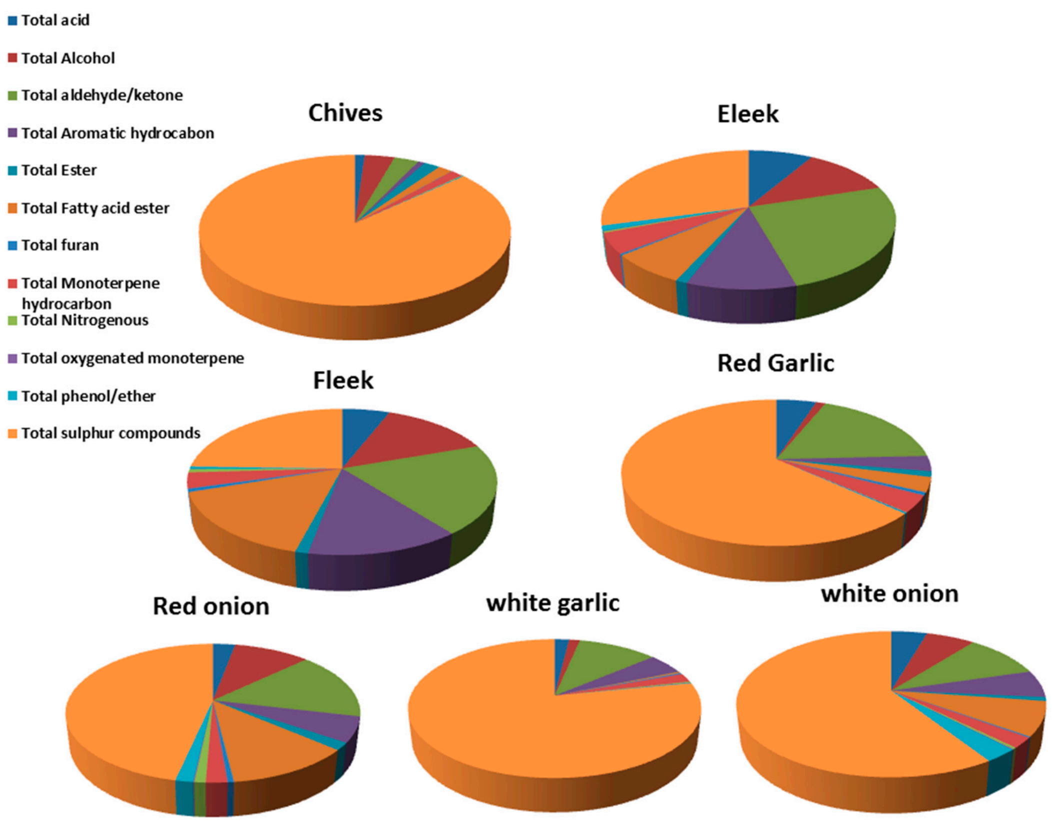

Figure S4. Pie chart illustrating distribution of different volatile classes identified in seven *Allium* species.

Table S1. Relative percentage of volatile metabolites in *Allium* species aerial parts analyzed via GC–MS, n = 3.

Peak number	Average Rt(min)	Average RI	Metabolite Name	Class	Chives		Egyptian Leek		French Leek		Red Garlic		Red Onion		White Garlic		White Onion	
					Av	sd	Av	sd	Av	sd	Av	sd	Av	sd	Av	sd	Av	sd
5	4.68	765.54	Isovaleric acid	Acid	0.03	0.03	0.00	0.00	0.03	0.04	0.04	0.03	0.00	0.00	0.15	0.12	0.00	0.00
21	5.79	873.08	Tiglic acid	Acid	0.01	0.01	0.00	0.00	0.00	0.00	0.05	0.08	0.04	0.02	0.02	0.02	0.01	0.00
29	6.49	938.79	Pentanoic acid	Acid	0.42	0.21	5.31	0.76	3.09	2.09	0.33	0.23	0.82	0.40	0.69	0.42	0.91	0.54
31	6.53	943.77	Valeric acid	Acid	0.05	0.03	0.12	0.10	0.37	0.24	0.18	0.09	0.13	0.06	0.06	0.01	0.06	0.04
57	7.68	1050.81	Heptanoic acid	Acid	0.13	0.13	0.74	0.78	0.45	0.13	3.60	6.21	0.18	0.14	0.30	0.18	2.31	4.27
77	8.65	1141.37	Benzoic acid	Acid	0.23	0.07	1.14	0.35	0.95	0.40	0.32	0.44	0.78	0.16	0.30	0.11	0.45	0.13
79	8.71	1147.64	Caprylic acid	Acid	0.12	0.05	0.41	0.21	0.47	0.51	0.14	0.16	0.19	0.11	0.11	0.05	0.18	0.07
96	9.69	1243.32	Nonanoic acid	Acid	0.08	0.02	0.53	0.41	0.39	0.25	0.18	0.18	0.25	0.14	0.16	0.04	0.17	0.07
115	10.59	1339.57	n-Capric acid	Acid	0.04	0.01	0.20	0.12	0.19	0.11	0.16	0.16	0.55	0.10	0.13	0.02	0.44	0.19
**Total acids**					**1.11**	**0.56**	**8.45**	**2.74**	**5.94**	**3.77**	**5.01**	**7.59**	**2.94**	**1.13**	**1.91**	**0.98**	**4.54**	**5.31**
3	4.58	756.32	2-Methylpentanol	Alcohol	0.04	0.02	2.72	0.43	0.11	0.09	0.01	0.00	0.10	0.03	0.03	0.02	0.15	0.10
7	4.85	782.77	3-Hexenol	Alcohol	1.18	0.44	2.26	1.18	2.57	2.42	0.56	0.45	0.71	0.39	0.70	0.63	1.60	1.26
10	4.93	791.07	3-Hexen-1-ol	Alcohol	0.01	0.00	0.09	0.07	0.09	0.12	0.02	0.01	0.02	0.01	0.01	0.01	0.01	0.01
12	5.08	806.51	2-Hexenol	Alcohol	0.32	0.08	1.02	0.25	1.02	0.76	0.07	0.01	0.75	0.50	0.40	0.33	0.52	0.16
13	5.13	810.26	n-Hexanol	Alcohol	0.03	0.02	0.06	0.02	0.19	0.05	0.03	0.02	0.05	0.00	0.03	0.03	0.12	0.13
35	6.61	950.69	Amyl vinyl carbinol	Alcohol	0.21	0.07	0.20	0.11	0.10	0.04	0.04	0.00	0.13	0.09	0.03	0.03	0.26	0.41
50	7.28	1013.80	Benzyl alcohol	Alcohol	0.18	0.14	0.24	0.11	0.98	0.88	0.20	0.05	0.31	0.24	0.11	0.18	0.19	0.20
70	8.23	1102.58	Benzyl Carbinol	Alcohol	0.30	0.05	2.54	0.32	0.79	0.49	0.07	0.05	0.19	0.06	0.11	0.01	0.44	0.20
139	15.15	1810.23	Phytol	Alcohol	0.62	0.41	1.36	1.12	4.39	1.98	0.06	0.06	4.82	1.60	0.00	0.00	1.07	0.80
143	15.35	1831.70	3,7,11,15-Tetramethyl-2-hexadecen-1-ol	Alcohol	0.19	0.12	0.44	0.31	1.30	0.59	0.03	0.02	1.06	0.69	0.00	0.00	0.29	0.23
144	15.51	1848.31	11-Hexadecenol	Alcohol	0.32	0.20	0.70	0.59	2.14	1.01	0.07	0.09	1.85	1.29	0.00	0.00	0.50	0.42
**Total alcohol**					**3.39**	**1.56**	**11.63**	**4.53**	**13.67**	**8.44**	**1.16**	**0.78**	**9.98**	**4.90**	**1.43**	**1.23**	**5.16**	**3.92**
1	4.50	747.14	2-Methyl-2-pentenal	Aldehyde/ketone	0.05	0.01	1.08	0.81	1.69	1.50	0.47	0.73	6.73	2.31	0.33	0.27	1.12	1.01
2	4.50	748.05	2-Hexenal	Aldehyde/ketone	0.01	0.00	0.10	0.04	0.05	0.06	0.09	0.15	0.04	0.01	0.05	0.03	0.98	1.82
6	4.81	779.89	2-Hexenal	Aldehyde/ketone	0.25	0.15	1.23	0.51	1.36	0.91	0.51	0.53	1.12	0.58	0.20	0.15	0.70	0.52
9	4.92	790.83	2-Butanal	Aldehyde/ketone	0.10	0.09	0.11	0.15	0.18	0.10	0.11	0.07	0.17	0.08	0.14	0.11	0.09	0.05
19	5.67	861.07	Sorbaldehyde	Aldehyde/ketone	0.00	0.00	0.07	0.07	0.00	0.00	0.38	0.31	0.14	0.23	0.91	1.58	0.12	0.21
27	6.32	922.72	Benzeneacetaldehyde	Aldehyde/ketone	0.01	0.01	0.16	0.12	0.04	0.05	0.14	0.12	0.08	0.02	0.39	0.08	0.04	0.05
28	6.38	928.81	Benzaldehyde	Aldehyde/ketone	0.19	0.02	10.18	3.01	3.89	1.46	0.10	0.05	0.39	0.11	0.29	0.03	1.30	0.43
39	6.71	958.98	Sulcatone	Aldehyde/ket	0.02	0.01	0.15	0.07	0.25	0.28	0.04	0.04	0.07	0.05	0.03	0.02	0.04	0.01

				one														
42	6.84	973.02	3,4-Dimethyl-2-cyclopenten-1-one	Aldehyde/ketone	0.50	0.11	1.75	0.23	3.49	2.80	0.78	0.93	1.20	0.41	0.47	0.33	0.77	0.17
45	7.00	987.49	2,4-Heptadienal	Aldehyde/ketone	0.22	0.03	0.44	0.04	0.40	0.20	0.08	0.03	0.18	0.07	0.08	0.03	0.23	0.07
46	7.18	1004.19	Melilotal	Aldehyde/ketone	0.01	0.00	0.82	0.49	1.34	0.46	0.03	0.00	0.21	0.05	0.03	0.01	0.26	0.09
51	7.42	1026.71	Benzeneacetaldehyde	Aldehyde/ketone	0.05	0.02	1.21	0.30	0.42	0.10	0.03	0.02	0.21	0.22	0.09	0.02	0.16	0.06
53	7.52	1036.22	p-Tolyl-acetaldehyde	Aldehyde/ketone	0.04	0.01	0.07	0.01	0.12	0.06	1.40	2.41	0.14	0.08	0.08	0.00	0.10	0.03
54	7.56	1039.49	2-Octenal	Aldehyde/ketone	0.03	0.01	0.36	0.22	0.47	0.06	7.79	13.49	0.10	0.01	0.05	0.03	0.14	0.05
58	7.71	1054.07	3,5-Octadien-2-one	Aldehyde/ketone	0.13	0.07	2.13	0.14	0.38	0.10	0.04	0.01	0.52	0.12	0.23	0.07	0.40	0.20
62	7.98	1078.44	3,5-Octadien-2-one, isomer	Aldehyde/ketone	0.09	0.04	2.77	0.54	0.25	0.08	0.06	0.06	0.35	0.11	0.28	0.09	0.75	0.25
67	8.11	1089.84	Nonanal	Aldehyde/ketone	0.37	0.12	0.77	0.26	1.25	1.19	3.69	2.96	0.68	0.17	5.43	0.43	1.52	0.72
76	8.55	1132.69	4-Ketoisophorone	Aldehyde/ketone	0.10	0.04	0.08	0.01	0.09	0.11	0.02	0.03	0.06	0.01	0.03	0.01	0.03	0.01
82	8.89	1163.84	4-Methylpropiophenone	Aldehyde/ketone	0.01	0.00	0.21	0.03	0.22	0.03	0.03	0.02	0.09	0.01	0.04	0.01	0.08	0.01
88	9.16	1188.66	Decanal	aldehyde/ketone	0.01	0.01	0.08	0.04	1.40	2.24	0.05	0.05	0.09	0.07	0.03	0.00	0.05	0.03
94	9.41	1213.49	β-Cyclocitral	aldehyde/ketone	0.22	0.02	0.50	0.04	0.61	0.13	0.04	0.00	1.32	1.97	0.25	0.26	0.18	0.05
97	9.72	1247.15	.p-Anisaldehyde	aldehyde/ketone	0.06	0.02	0.20	0.02	0.30	0.09	0.34	0.16	0.25	0.16	0.15	0.18	0.11	0.07
98	9.82	1257.92	Myrtenal	aldehyde/ketone	0.01	0.00	0.14	0.05	0.05	0.03	0.03	0.06	0.17	0.02	0.01	0.01	0.17	0.04
100	10.01	1277.28	2-Undecanone	Aldehyde/ketone	0.20	0.15	0.45	0.35	1.11	0.66	1.69	1.44	0.94	0.81	0.66	1.03	0.31	0.27
116	10.68	1348.21	2-Undecenal	Aldehyde/ketone	0.04	0.02	0.17	0.11	0.27	0.19	0.03	0.02	0.15	0.13	0.11	0.09	0.10	0.03
118	10.92	1373.10	Anisic ketone	aldehyde/ketone	0.02	0.01	0.04	0.02	0.07	0.04	0.05	0.03	0.19	0.08	0.04	0.03	0.05	0.02
119	11.05	1387.11	Vanillin	aldehyde/ketone	0.04	0.01	0.07	0.01	0.03	0.01	0.01	0.02	0.04	0.04	0.01	0.01	0.02	0.01
120	11.06	1388.11	2-Hydroxy-4-methoxybenzaldehyde	aldehyde/ketone	0.02	0.00	0.16	0.02	0.10	0.02	0.05	0.08	0.16	0.05	0.08	0.08	0.08	0.03
125	11.83	1463.43	2-Tridecanone	Aldehyde/ket	0.02	0.00	0.09	0.03	0.32	0.02	0.07	0.08	0.07	0.04	0.05	0.02	0.05	0.02

				one														
126	11.86	1466.27	β-Ionone	Aldehyde/ketone	0.11	0.02	1.32	0.17	1.77	0.56	0.04	0.02	0.26	0.05	0.22	0.06	0.58	0.12
140	15.20	1816.13	Perhydrofarnesyl acetone	Aldehyde/ketone	0.30	0.14	0.60	0.09	0.88	0.02	0.08	0.06	1.56	1.07	0.00	0.00	0.36	0.25
		Total aldehyde/ketone			**3.25**	**1.13**	**27.53**	**8.01**	**22.80**	**13.55**	**18.27**	**23.99**	**17.67**	**9.13**	**10.74**	**5.06**	**10.89**	**6.70**
65	8.02	1083.24	Undecane	Aliphatic hydrocarbon	0.05	0.01	0.11	0.01	0.13	0.04	1.02	0.84	0.18	0.01	1.46	0.20	1.07	1.70
86	9.10	1182.97	Dodecane	Aliphatic hydrocarbon	0.03	0.01	0.09	0.03	0.23	0.04	1.52	0.44	0.25	0.11	2.37	1.08	0.08	0.02
89	9.24	1196.64	.2,6-Dimethylundecane	Aliphatic hydrocarbon	0.00	0.00	0.06	0.01	0.15	0.19	0.04	0.02	0.04	0.01	0.10	0.04	0.04	0.01
		**Total aliphatic hydrocarbon**			**0.08**	**0.03**	**0.26**	**0.04**	**0.51**	**0.27**	**2.59**	**1.30**	**0.47**	**0.14**	**3.93**	**1.33**	**1.19**	**1.72**
11	5.07	804.54	p-Xylol	Aromatic hydrocabon	0.01	0.01	0.44	0.37	0.54	0.78	0.02	0.01	0.11	0.05	0.03	0.03	0.13	0.10
16	5.43	838.46	p-Xylene	Aromatic hydrocabon	0.00	0.00	0.00	0.00	0.00	0.00	0.01	0.01	0.00	0.00	0.02	0.01	0.00	0.00
17	5.49	844.67	m-Xylene	Aromatic hydrocabon	0.00	0.00	0.01	0.01	0.00	0.00	0.02	0.02	0.04	0.02	0.04	0.05	0.01	0.01
40	6.80	968.48	Hemellitol	Aromatic hydrocabon	0.02	0.01	0.09	0.02	0.95	0.67	0.01	0.01	0.16	0.02	0.03	0.02	0.05	0.04
41	6.82	970.83	Pseudocumene	Aromatic hydrocabon	0.03	0.02	0.73	0.05	1.94	1.33	0.21	0.25	0.32	0.03	0.22	0.05	0.26	0.17
48	7.19	1005.02	Cumene	Aromatic hydrocabon	0.00	0.00	0.00	0.00	0.00	0.00	0.00	0.00	0.00	0.00	0.00	0.00	0.05	0.10
61	7.91	1072.50	m-Xylene, 2-ethyl-	Aromatic hydrocabon	0.03	0.02	0.70	0.27	0.89	0.24	0.15	0.09	0.14	0.02	0.01	0.00	0.21	0.12
78	8.68	1143.87	Isodurene	Aromatic hydrocabon	0.02	0.01	1.07	0.26	0.08	0.02	0.01	0.00	1.34	0.38	0.03	0.01	1.10	0.66
84	9.05	1178.82	Naphthalene	Aromatic hydrocabon	0.10	0.03	3.73	0.28	4.69	1.43	0.18	0.06	0.97	0.22	0.20	0.04	1.36	0.82
99	9.86	1262.83	.5-Methyltetralin	Aromatic hydrocabon	0.00	0.00	0.12	0.07	0.04	0.03	0.02	0.02	0.20	0.05	0.02	0.01	0.19	0.04
106	10.16	1292.94	β-Methylnaphthalene	Aromatic hydrocabon	0.03	0.01	1.00	0.20	0.90	0.23	0.03	0.01	0.24	0.03	0.03	0.00	0.53	0.11
		**Total aromatic hydrocarbon**			**0.25**	**0.10**	**7.87**	**1.53**	**10.03**	**4.73**	**0.64**	**0.48**	**3.52**	**0.82**	**0.65**	**0.23**	**3.90**	**2.17**
44	6.94	981.88	4-Hexenyl acetate	Ester	0.68	0.35	0.77	0.37	0.49	0.23	0.17	0.13	0.90	0.42	0.08	0.08	0.67	0.17
137	14.68	1759.43	Benzyl Benzoate	Ester	1.18	0.29	0.34	0.06	0.64	0.04	1.07	0.63	0.84	0.65	0.00	0.00	0.24	0.13
		**Total ester**			**1.86**	**0.64**	**1.11**	**0.43**	**1.14**	**0.27**	**1.25**	**0.76**	**1.74**	**1.07**	**0.08**	**0.08**	**0.91**	**0.30**
122	11.28	1410.18	Decanoic acid, methyl ester	Fatty acid/ester	0.05	0.02	0.12	0.02	0.81	0.71	0.37	0.51	0.62	0.15	0.21	0.23	0.29	0.05
127	12.11	1491.33	Lauric acid, methyl	Fatty	0.04	0.01	0.02	0.01	0.04	0.06	0.01	0.02	0.04	0.01	0.04	0.04	0.02	0.01

			ester	acid/ester														
129	12.43	1521.84	Dodecanoic acid	Fatty acid/ester	0.09	0.04	0.39	0.11	0.59	0.52	0.12	0.16	0.21	0.07	0.09	0.10	0.29	0.18
135	14.26	1712.60	Methyl tridecanoate	Fatty acid/ester	0.01	0.01	0.07	0.04	0.12	0.10	0.01	0.01	0.39	0.64	0.00	0.00	0.01	0.00
136	14.48	1737.80	Myristic acid	Fatty acid/ester	0.18	0.05	1.03	0.62	1.68	1.30	0.33	0.31	1.15	0.74	0.00	0.00	0.79	0.43
141	15.23	1818.39	Tetradecanoic acid	Fatty acid/ester	0.09	0.03	0.44	0.22	0.75	0.59	0.01	0.01	0.16	0.19	0.00	0.00	0.59	0.94
142	15.28	1823.93	Pentadecanoic acid	Fatty acid/ester	0.06	0.01	0.30	0.18	0.68	0.85	0.20	0.18	0.25	0.10	0.00	0.00	0.25	0.13
145	15.89	1887.45	Palmitic acid, methyl ester	Fatty acid/ester	0.21	0.08	0.52	0.04	0.46	0.11	0.13	0.11	0.21	0.04	0.00	0.00	0.22	0.03
146	15.99	1898.11	Hexadecenoic acid	Fatty acid/ester	0.00	0.00	0.01	0.01	0.04	0.05	0.00	0.00	0.06	0.07	0.00	0.00	0.02	0.00
147	16.01	1899.71	Palmitoleic acid	Fatty acid/ester	0.05	0.02	0.46	0.25	1.38	1.88	0.18	0.11	0.40	0.21	0.00	0.00	0.43	0.29
148	16.20	1919.61	Palmitic acid	Fatty acid/ester	0.90	0.21	3.79	0.61	9.20	6.64	1.85	2.24	8.27	2.77	0.00	0.00	4.74	2.70
**Total Fatty acid/ester**					**1.68**	**0.48**	**7.17**	**2.09**	**15.76**	**12.80**	**3.20**	**3.64**	**11.76**	**4.97**	**0.34**	**0.38**	**7.64**	**4.75**
92	9.37	1209.85	Hydroxymethylfurfural	Furan	0.06	0.01	0.26	0.04	0.76	0.61	0.64	0.91	0.53	0.30	0.29	0.10	0.23	0.12
132	13.14	1591.28	2-Octanoylfuran	Furan	0.00	0.00	0.07	0.01	0.01	0.02	0.01	0.00	0.05	0.05	0.03	0.06	0.02	0.01
**Total furan**					**0.06**	**0.01**	**0.33**	**0.05**	**0.77**	**0.63**	**0.65**	**0.92**	**0.59**	**0.35**	**0.33**	**0.15**	**0.25**	**0.13**
24	6.02	894.98	.β-Thujene	Monoterpene hydrocarbon	0.02	0.01	0.03	0.04	0.01	0.01	0.02	0.01	0.07	0.06	0.01	0.01	0.01	0.01
32	6.55	945.08	α-Phellandrene	Monoterpene hydrocarbon	0.21	0.06	1.90	0.25	0.59	0.43	0.37	0.58	0.27	0.03	0.21	0.16	0.60	0.21
34	6.60	949.28	β-Pinene	Monoterpene hydrocarbon	0.41	0.17	0.67	0.13	0.78	0.39	0.21	0.27	0.12	0.08	0.16	0.11	0.79	0.62
47	7.18	1004.36	o-Cymene	Monoterpene hydrocarbon	0.12	0.03	0.57	0.18	0.93	0.10	0.06	0.02	0.17	0.09	0.05	0.01	0.14	0.05
49	7.25	1010.98	Limonene	Monoterpene hydrocarbon	0.41	0.17	0.69	0.84	0.09	0.00	0.24	0.19	0.64	1.02	0.18	0.15	0.16	0.14
52	7.46	1030.00	α-Ocimene	Monoterpene hydrocarbon	0.00	0.00	0.10	0.01	0.04	0.01	0.82	1.40	0.13	0.03	0.01	0.01	0.07	0.04
55	7.59	1042.40	γ-Terpinene	Monoterpene hydrocarbon	0.07	0.04	0.43	0.01	0.48	0.04	0.11	0.12	0.11	0.09	0.08	0.06	0.14	0.03
56	7.60	1043.74	α-Phellandrene	Monoterpene hydrocarbon	0.11	0.04	0.21	0.27	0.12	0.09	0.10	0.06	0.18	0.22	0.08	0.05	0.13	0.06
59	7.85	1067.48	o-Cymene	Monoterpene hydrocarbon	0.05	0.02	0.19	0.05	0.45	0.16	1.75	0.50	0.29	0.12	1.29	0.03	0.30	0.07

**Total monoterpene hydrocarbon**					**1.40**	**0.55**	**4.78**	**1.77**	**3.49**	**1.24**	**3.69**	**3.16**	**1.96**	**1.74**	**2.08**	**0.60**	**2.33**	**1.23**
103	10.10	1286.82	Indole	Nitrogenos	0.12	0.06	0.30	0.18	0.74	0.21	0.06	0.06	1.02	0.57	0.25	0.18	0.33	0.18
**Total nitrogenous**					**0.12**	**0.06**	**0.30**	**0.18**	**0.74**	**0.21**	**0.06**	**0.06**	**1.02**	**0.57**	**0.25**	**0.18**	**0.33**	**0.18**
102	10.05	1281.81	Safrole	Phenol/ether	0.02	0.00	0.11	0.02	0.00	0.01	0.01	0.01	0.03	0.00	0.04	0.00	0.11	0.10
104	10.11	1287.83	Carvacrol	Phenol/ether	0.00	0.00	0.01	0.01	0.02	0.01	0.07	0.05	0.07	0.03	0.03	0.03	0.01	0.01
**Total Phenol/ether**					**0.02**	**0.00**	**0.12**	**0.03**	**0.02**	**0.02**	**0.08**	**0.06**	**0.10**	**0.03**	**0.07**	**0.03**	**0.12**	**0.10**
87	9.13	1186.47	Estragole	Oxygenated monoterpene	0.09	0.02	0.04	0.01	0.16	0.27	0.11	0.03	0.05	0.01	0.02	0.01	0.04	0.02
91	9.34	1206.77	Arosol	Oxygenated monoterpene	0.03	0.01	0.53	0.59	0.22	0.14	0.04	0.01	0.36	0.06	0.04	0.01	0.11	0.04
101	10.01	1277.70	Anethole	Oxygenated monoterpene	0.02	0.01	0.63	0.05	0.22	0.16	0.06	0.01	1.22	0.39	0.08	0.01	2.89	0.83
**Total oxygenated**					**0.14**	**0.04**	**1.20**	**0.65**	**0.60**	**0.57**	**0.21**	**0.06**	**1.64**	**0.46**	**0.14**	**0.03**	**3.05**	**0.89**
4	4.65	762.69	1,2-Dithiolane	Sulfur compound	0.02	0.01	2.75	0.40	0.13	0.09	0.02	0.02	0.07	0.05	0.03	0.01	0.17	0.11
8	4.89	786.56	Allyl sulfide	Sulfur compound	2.08	0.81	2.68	0.81	2.78	2.63	0.52	0.45	1.78	0.40	0.36	0.20	0.65	0.48
14	5.21	818.26	2,4-Dimethylthiophene	Sulfur compound	0.04	0.01	0.30	0.05	0.15	0.13	0.02	0.01	0.08	0.01	0.02	0.01	0.22	0.14
15	5.34	829.86	di(1-Propenyl) sulfide	Sulfur compound	0.02	0.00	0.06	0.02	0.09	0.05	0.02	0.01	0.04	0.02	0.06	0.02	0.06	0.08
18	5.59	854.18	3,4-Dimethylthiophene	Sulfur compound	0.04	0.01	0.06	0.04	0.08	0.10	0.03	0.01	0.09	0.08	0.10	0.10	0.02	0.01
20	5.77	871.04	Allyl methyl disulfide	Sulfur compound	0.03	0.01	0.11	0.02	0.01	0.01	0.38	0.32	0.38	0.11	1.62	1.84	0.41	0.21
22	5.87	880.07	2,5-Dimethylthiophene	Sulfur compound	0.08	0.07	3.83	1.19	0.28	0.12	0.17	0.19	0.14	0.05	0.08	0.03	1.40	0.89
23	5.98	891.15	Methyl propyl disulfide	Sulfur compound	0.01	0.00	0.39	0.06	0.11	0.16	0.01	0.01	0.52	0.14	0.23	0.09	0.28	0.14
25	6.10	901.88	Methyl propenyl disulfide	Sulfur compound	0.01	0.01	0.41	0.06	0.05	0.02	0.08	0.09	0.44	0.11	0.18	0.18	0.17	0.10
26	6.10	902.28	Allyl methyl disulfide	Sulfur compound	0.00	0.00	0.08	0.03	0.01	0.02	0.00	0.00	0.02	0.02	0.01	0.00	0.02	0.02
30	6.52	941.58	Methyl trisulfide	Sulfur compound	0.05	0.01	0.55	0.07	0.53	0.10	0.10	0.04	0.13	0.08	0.12	0.03	0.20	0.07
33	6.60	949.64	Dimethyldisulfide, S-oxide	Sulfur compound	0.95	0.42	0.95	0.19	1.08	0.81	0.07	0.07	0.12	0.08	0.10	0.08	0.27	0.21
36	6.62	951.62	.2-Methyl-1,3-dithiolane	Sulfur compound	0.12	0.05	0.21	0.04	0.28	0.26	0.07	0.10	0.11	0.04	0.06	0.03	0.27	0.07
37	6.67	956.18	.2,4-Dimethylthiazole	Sulfur compound	0.05	0.02	0.49	0.24	2.53	2.11	0.03	0.01	0.26	0.06	0.04	0.01	0.18	0.08
38	6.67	956.29	3-Butenyl	Sulfur	0.00	0.00	0.00	0.00	0.00	0.00	0.00	0.00	0.00	0.00	0.00	0.00	0.02	0.04

			isothiocyanate	compound														
43	6.92	979.31	2-Propyl-thiolane	Sulfur compound	0.02	0.02	0.17	0.07	0.05	0.03	0.04	0.01	0.18	0.07	0.09	0.03	0.15	0.07
60	7.85	1067.17	Diallyl disulphide	Sulfur compound	0.56	0.26	1.45	1.20	0.23	0.10	36.76	23.62	2.58	1.65	32.79	0.76	4.77	2.61
63	8.00	1082.09	2-Ethyl[1,3]dithiane	Sulfur compound	0.00	0.00	0.01	0.00	0.00	0.01	0.42	0.31	0.06	0.01	0.13	0.02	3.84	7.54
64	8.01	1082.04	1,2-Dithiolane	Sulfur compound	0.14	0.06	0.73	0.08	0.91	0.10	3.79	2.94	0.74	0.08	2.30	0.34	1.24	0.90
66	8.09	1089.17	Diallyl disulphide	Sulfur compound	0.08	0.03	0.16	0.08	0.15	0.18	3.91	3.28	0.39	0.06	6.03	0.44	1.20	0.58
68	8.14	1094.32	Allyl disulfide	Sulfur compound	0.07	0.03	0.26	0.08	0.70	0.32	0.15	0.17	0.16	0.03	0.11	0.02	0.12	0.03
69	8.17	1096.18	Propyl disulfide	Sulfur compound	0.15	0.05	3.60	1.07	5.72	5.49	0.26	0.04	18.40	4.35	0.10	0.03	18.91	12.65
71	8.25	1104.41	Ethyl methylthiopropanoate	Sulfur compound	0.01	0.01	0.01	0.00	0.01	0.01	0.04	0.07	0.01	0.00	0.00	0.00	0.04	0.06
72	8.26	1104.69	2,2-Dimethyl-1,3-dithiane	Sulfur compound	0.01	0.01	0.44	0.08	0.53	0.47	0.16	0.14	3.19	0.59	0.11	0.02	2.49	1.68
73	8.38	1116.03	2-Vinyl-1,3-dithiane	Sulfur compound	0.02	0.02	0.11	0.08	0.17	0.26	0.11	0.15	0.07	0.07	0.07	0.05	0.07	0.04
74	8.43	1121.39	2,4,5-Trithiahexane	Sulfur compound	13.59	2.83	0.19	0.09	0.16	0.10	0.01	0.00	0.00	0.00	0.01	0.00	0.02	0.03
75	8.54	1131.28	Allyl methyl trisulfide	Sulfur compound	0.04	0.01	0.27	0.04	0.10	0.05	0.20	0.11	0.36	0.08	3.42	0.31	0.09	0.05
80	8.79	1154.33	S-1-Propenylmethanethiosulfonate	Sulfur compound	0.00	0.00	0.05	0.04	0.03	0.03	0.15	0.24	0.09	0.06	0.04	0.02	0.26	0.47
81	8.83	1157.67	Methyl propenyl sulfide	Sulfur compound	0.08	0.02	0.08	0.02	0.08	0.06	0.05	0.07	0.16	0.03	0.03	0.01	0.04	0.00
83	8.94	1168.82	2-Mercapto-3,4-dimethyl-2,3-dihydrothiophene	Sulfur compound	0.02	0.00	0.19	0.02	0.19	0.06	0.52	0.31	0.97	0.22	0.45	0.06	0.65	0.36
85	9.07	1180.35	3-Vinyl-1,2-dithiacyclohex-4-ene	Sulfur compound	0.15	0.03	0.07	0.02	0.10	0.11	0.01	0.02	0.08	0.02	0.02	0.01	0.04	0.03
90	9.34	1206.38	3-Vinyl-1,2-dithiacyclohex-5-ene	Sulfur compound	0.04	0.00	0.11	0.02	0.19	0.08	3.65	2.96	0.10	0.10	7.08	2.58	0.09	0.11
93	9.39	1211.92	Dimethyl tetrasulphide	Sulfur compound	0.17	0.02	0.67	0.14	0.63	0.15	0.68	0.37	0.75	0.58	0.10	0.02	0.22	0.02
95	9.46	1219.24	Benzothiazol	Sulfur compound	0.04	0.01	0.24	0.07	0.67	0.11	0.08	0.10	0.33	0.33	0.05	0.01	0.30	0.20
105	10.13	1290.06	2,4,5-Trithiahexane	Sulfur	0.01	0.00	0.03	0.01	0.06	0.03	0.05	0.08	0.03	0.01	0.04	0.00	0.04	0.02

				compound														
107	10.18	1295.29	Allyl trisulfide	Sulfur compound	0.02	0.01	0.12	0.02	0.13	0.04	4.51	0.98	0.09	0.01	15.50	0.66	0.09	0.05
108	10.31	1309.09	Isobutyl isothiocyanate	Sulfur compound	0.01	0.00	0.27	0.08	0.09	0.02	0.33	0.20	0.18	0.02	0.26	0.02	0.30	0.03
109	10.32	1309.11	Propyl trisulfide	Sulfur compound	0.06	0.00	0.05	0.02	0.14	0.18	0.02	0.01	0.02	0.01	0.03	0.01	0.06	0.01
110	10.38	1316.76	3-Vinyl-1,2-dithi-4-ene	Sulfur compound	0.00	0.00	0.01	0.00	0.01	0.01	0.05	0.04	0.05	0.04	0.13	0.03	0.03	0.02
111	10.43	1321.60	Allyl sulfide	Sulfur compound	0.06	0.01	1.66	0.19	0.88	0.49	0.28	0.03	5.40	0.97	1.48	0.18	10.41	1.68
112	10.50	1328.93	3-Vinyl-1,2-dithiacyclohex-5-ene	Sulfur compound	0.00	0.00	0.13	0.04	0.04	0.02	0.11	0.12	0.27	0.07	0.09	0.03	0.30	0.04
113	10.56	1335.41	1,2,4-Trithiolane, 3,5-diethyl-	Sulfur compound	0.03	0.00	0.67	0.23	0.20	0.03	0.69	0.40	3.33	0.95	1.64	0.40	4.57	0.48
114	10.57	1336.25	Diallyl monosulfide	Sulfur compound	0.06	0.02	0.68	0.13	0.40	0.12	1.01	0.36	2.30	0.40	1.55	0.41	2.85	0.27
117	10.78	1359.02	Benzyl Isothiocyanate	Sulfur compound	0.20	0.21	0.87	0.68	1.57	1.93	1.30	2.23	0.56	0.78	0.49	0.44	0.99	0.94
121	11.14	1396.63	6-Methylthianaphthene	Sulfur compound	0.01	0.01	0.03	0.04	0.13	0.10	0.11	0.17	0.04	0.02	0.03	0.02	0.02	0.01
123	11.51	1433.22	2-Butyl-thiolane	Sulfur compound	0.02	0.00	0.04	0.01	0.08	0.03	0.10	0.06	0.38	0.58	0.10	0.04	0.02	0.01
124	11.70	1451.55	Phenethyl isothiocyanate	Sulfur compound	0.03	0.02	1.06	0.25	0.27	0.10	0.03	0.04	0.41	0.11	0.04	0.01	0.73	0.13
128	12.23	1502.82	**Tetrathiaoctane**	Sulfur compound	63.54	5.16	0.99	0.67	1.12	0.56	1.94	1.05	0.06	0.02	0.46	0.02	0.04	0.01
130	12.46	1525.07	Diallyl tetrasulphide	Sulfur compound	0.07	0.02	0.29	0.08	0.46	0.17	0.07	0.09	0.19	0.17	0.25	0.39	0.16	0.11
131	12.84	1561.60	1-Propyl-2-(4-thiohept-2-en-5-yl)disulfide	Sulfur compound	0.00	0.00	0.00	0.00	0.00	0.01	0.00	0.00	0.20	0.17	0.02	0.04	0.01	0.01
133	13.28	1605.65	2,4-Dimethyl-5,6-dithia-2,7-nonadienal	Sulfur compound	0.01	0.00	0.51	0.28	0.07	0.05	0.03	0.04	0.08	0.01	0.01	0.01	0.08	0.01
134	13.55	1634.64	2,4-Dimethyl-5,6-dithia-2,7-nonadienal	Sulfur compound	0.01	0.00	0.03	0.00	0.04	0.01	0.03	0.06	0.09	0.04	0.00	0.00	0.03	0.02
138	14.77	1768.69	2,4-Dimethyl-5,6-dithia-2,7-nonadienal	Sulfur compound	3.83	2.32	0.12	0.04	0.11	0.06	0.03	0.01	0.07	0.05	0.00	0.00	0.06	0.02
		**Total sulpher compound**			**86.62**	**12.64**	**29.24**	**9.21**	**24.54**	**18.29**	**63.19**	**42.21**	**46.58**	**14.00**	**78.06**	**10.13**	**59.68**	**33.98**

The results were represented in average and SD
